# Delays in screening, diagnosis and monitoring are associated with worse clinical and economic outcomes for patients with chronic kidney disease: a narrative review

**DOI:** 10.1080/07853890.2026.2753927

**Published:** 2026-11-11

**Authors:** Nicole Fusco, James Weatherall, Jayne Hurrell

**Affiliations:** ^a^ Cencora, Conshohocken, PA, USA; ^b^ Boehringer Ingelheim International GmbH, Ingelheim am Rhein, Germany; ^c^ Cencora, London, UK

**Keywords:** Chronic kidney disease, screening, diagnosis, healthcare costs, cost-effectiveness, adverse cardiovascular events

## Abstract

**Background:**

Chronic kidney disease (CKD) has substantial associated morbidity, mortality and healthcare costs that increase as CKD progresses. Despite newer, more effective treatments, the global burden of CKD continues to increase.

**Objective:**

To understand patterns of screening, diagnosis, monitoring and treatment in CKD and how earlier treatment initiation could help mitigate CKD burden.

**Methods:**

Targeted searches of bibliographic databases and grey literature sources identified studies published 2015–2025. Eligible evidence comprised observational studies on CKD screening and diagnosis rates, factors associated with delayed treatment initiation, clinical and economic burden of delayed diagnosis/treatment, and economic evaluations of population-based CKD screening. Thirty-five studies were synthesized narratively according to the CKD care pathway; no meta-analysis or formal quality assessment was conducted.

**Discussion:**

Despite guideline recommendations, 19%–33% of patients in at-risk populations received neither kidney function nor albuminuria testing. Documented CKD diagnosis rates were often low, particularly in earlier stages. Documented CKD diagnosis was associated with increased monitoring and prescription fill rates, as well as slower decline of estimated glomerular filtration rate. Longer time to documented diagnosis and nephrology referral timing were associated with increased risk of kidney failure and cardiovascular events. Recent economic models that incorporated newer treatments and cardiovascular events generally produced found selected population-based screening strategies cost-effective. However, findings varied by target population, screening strategy/frequency, treatment, model assumptions, setting and willingness-to-pay threshold, and not all modelled scenarios were cost-effective.

**Conclusions:**

Results from observational studies suggest an association between earlier CKD diagnosis and greater use of appropriate monitoring and nephrology referral, which may facilitate timely intervention to slow progression, improving long-term outcomes. However, many patients remain undiagnosed despite available laboratory data. Improvements in risk-based testing, screening, diagnosis, monitoring and nephrology referral may help patients access effective treatments, but stronger comparative evidence is needed to establish their effects on clinical outcomes and costs.

## KEY MESSAGES

- Although documented CKD diagnosis and nephrology referral have been associated with more favourable clinical and economic outcomes in observational studies, the proportion with a documented CKD diagnosis was generally low, and many patients did not receive timely referral to a nephrologist.
- Recent economic models suggest that population-wide screening for CKD may be cost-effective in some settings and scenarios, depending on the population, screening strategy and frequency, treatment assumptions and willingness-to-pay thresholds and may improve kidney and cardiovascular outcomes.
- For patients to reap the full benefits of newer, more effective treatments for CKD, substantial improvements are needed in albuminuria testing, clinical diagnosis, disease monitoring and referral to nephrologists.

## Introduction

Chronic kidney disease (CKD) is a progressive disease that may eventually lead to kidney failure [1]. Early stages of CKD (i.e. stages 1–3) are largely asymptomatic or include only nonspecific symptoms such as fatigue, meaning that many patients are not screened or diagnosed until later stages of disease when additional symptoms begin to appear or they develop related comorbid conditions or complications. After diagnosis, patients with CKD require ongoing disease management. The Kidney Disease: Improving Global Outcomes (KDIGO) guidelines recommend that patients with CKD should have both creatinine and albuminuria assessed at least annually, with more frequent monitoring for advanced disease and should be referred to a nephrologist if their estimated glomerular filtration rate (eGFR) is <30 mL/min/1.73 m^2^ (i.e. GFR categories G4–G5), albumin-to-creatinine ratio (ACR) is ≥300 mg/g, or 5-year risk of kidney failure is 3% to 5% [1]. When patients reach kidney failure, they will require kidney replacement therapy (KRT) in the form of either dialysis (either peritoneal dialysis or haemodialysis) or a kidney transplant, unless they decide to pursue conservative therapy.

Common risk factors for CKD include hypertension, diabetes, cardiovascular diseases (CVDs) such as heart failure (HF), prior acute kidney injury (AKI) and family history, and the KDIGO guidelines recommend routine CKD screening for these high-risk patients [1]. Better understanding of risk factors for CKD provides an opportunity for intervention.

Although effective treatment is crucial to improving clinical and economic outcomes in patients with CKD, it is not enough in isolation. Despite the approval of effective CKD treatments such as sodium-glucose cotransporter 2 (SGLT-2) inhibitors and nonsteroidal mineralocorticoid receptor antagonists (MRAs), CKD continues to represent a substantial clinical and economic burden, particularly in terms of cardiovascular outcomes and kidney failure, and this burden is projected to continue increasing [2]. In 2017, nearly 700 million people worldwide had CKD, a 29.3% increase since 1990 [3]. Similarly, the annual incidence of dialysis and kidney transplant increased by 43.1% and 34.4%, respectively, in the same time period [3]. Nearly 2.4 million people cross eight countries (Australia, Brazil, China, Germany, Netherlands, Spain, United Kingdom and United States [US]) were either receiving dialysis or living post-transplant in 2022; this number is projected to double by 2032 [2]. Moreover, more than 1.2 million people died due to CKD in 2017, a 41.5% increase since 1990, including a 33.7% increase since 2007 [3,4]. This increased burden of CKD is projected to continue to increase, with 3.1 million deaths estimated in 2040, bringing CKD to the fifth leading cause of death worldwide [5]. In addition, CKD increases risk of death due to other causes. Of the 17.8 million deaths due to CVD, 1.4 million (7.8%) were attributable to impaired kidney function [3,4]. Depending on the country, myocardial infarction events and stroke events are projected to increase by 5.7%–41.7% and 26.0%–73.5%, respectively, among people with CKD between 2022 and 2032 [2]. Furthermore, the trajectory of overall CKD burden lags behind other noncommunicable diseases. Between 1990 and 2007, the age-standardized years of life lost improved substantially for ischaemic heart disease (20.2%), stroke (24.0%) and chronic obstructive pulmonary disease (COPD) (37.4%), while only a slight improvement was observed for CKD (7.2%) [4]. This trend continued between 2007 and 2017, with larger improvements seen for ischaemic heart disease (9.8%), stroke (13.8%) and COPD (14.3%) than CKD (2.5%) [4]. Similar results were seen in analyses of disability-adjusted life-years for CKD compared with ischaemic heart disease, stroke and COPD [6].

CKD represents an extensive burden beyond mortality. As CKD progresses, patients develop new symptoms of CKD [7], as well as additional complications, such as anaemia [8–10], mineral bone disorders [11–13], hyperkalaemia [12,14,15], fractures [16,17] and infections [10,18]. Some of these complications also contribute additional risk for more severe clinical outcomes, such as hospitalization, major adverse cardiovascular events and death [8–11,16–20]. More advanced CKD requires more treatment for both underlying disease and the higher frequency of complications, leading to higher medical and pharmacy costs [14,21–26]. Even though costs are higher at later stages of CKD, healthcare costs are higher for early-stage CKD than in the general population [14,21–23]. Among patients with early-stage CKD (i.e. stages 1–3), increased inpatient care needs are a major driver of increased healthcare costs, making up anywhere from 39% to 81% of total healthcare costs [14,21,22,25,26]. Among patients with kidney failure who require KRT, dialysis is the primary cost driver. In 2020, US Medicare expenditures for dialysis totalled more than $30 billion, accounting for almost 80% of Medicare spending on patients with kidney failure, and private insurance generally pays significantly more for dialysis per person per month than Medicare [27].

It is also important to consider that there is an extensive economic burden of CKD beyond direct healthcare costs. CKD can have a significant impact on patients’ mobility and physical functioning even in early stages, which becomes more pronounced as disease progresses [28–31]. Impaired functioning, as well as other factors such as time needed to attend medical visits and receive treatment, may affect patients’ work productivity. In a systematic review of 58 studies on employment in patients with kidney failure, the weighted mean employment rate before dialysis was 59.0%; during dialysis, the weighted mean employment rate was only 26.3% [32]. Among patients with kidney failure who received transplants, pre-transplant employment was 36.9%, while post-transplant employment was 38.2%. Work productivity may also be impacted among employed patients with CKD, including those not receiving dialysis. In a study of employed patients with CKD from the US, Europe and China, the average overall work impairment ranged from 22.1% in stage 3a to 35.8% in non-dialysis-dependent stage 5 CKD [31]. As CKD progression leads to additional functional impairments, patients with CKD require more assistance from caregivers, often in the form of transportation to medical appointments or help completing daily tasks. As many as two-thirds of patients with late-stage pre-dialysis CKD (stage 4–5) may require a caregiver, while nearly 40% require paid help with domestic work such as housekeeping [33].

Morbidity, mortality, quality of life, caregiver burden, costs and healthcare resource utilization all worsen as CKD progresses. Although the burden of CKD is multifactorial, this review focuses on potentially modifiable gaps along the identification-to-care pathway. This focus is particularly relevant because the availability of effective kidney- and cardiovascular-protective therapies does not ensure that eligible patients receive them: patients must first be identified, appropriately assessed, monitored and referred when indicated before treatment can be initiated or optimized. Gaps at one stage may therefore restrict opportunities for care at subsequent stages, although the observational evidence available to evaluate these relationships does not establish a causal sequence.

Risk-based testing refers to testing individuals selected because they have established CKD risk factors, such as diabetes, hypertension or cardiovascular disease, whereas population screening refers to systematic testing of an unselected general population. Population screening is considered separately in this review because it represents a broader identification strategy with distinct clinical, resource and policy implications; accordingly, its potential value is examined through available economic evaluations.

Against this background, this narrative review examines evidence on CKD identification and management across the care pathway, focusing on risk-based testing and population screening, documented CKD diagnosis, disease monitoring, nephrology care and receipt of guideline-recommended treatments. It also examines the clinical and economic outcomes associated with gaps in these areas and the modelled cost-effectiveness of CKD screening in the general population. These components are considered together to identify where gaps occur and how they may be associated with subsequent care and outcomes. Findings are interpreted according to study design and are not intended to establish that any individual gap causes the outcomes observed.

## Review methods

### Search strategy and information sources

A structured search of Embase *via* Ovid was conducted in January 2025 using both Emtree and free-text terms for CKD combined with terms relating to screening, diagnosis, epidemiology, treatment patterns, healthcare resource use and economic burden. Supplemental targeted searches were conducted in Embase, Medline, EconLit and the National Health Services Economic Evaluation Database (all *via* Ovid), as well as Google Scholar to capture studies that might fill data gaps identified in the primary search and to identify evidence published after the original search. Grey literature searches were conducted to identify reports by national bodies and registries (e.g. US Renal Data System, United Kingdom Kidney Association), as well as relevant guidelines. Finally, the citation lists of relevant systematic reviews were checked to identify additional relevant studies. The recorded search approaches are provided in Supplementary Table 1.

### Eligibility and study selection

Eligible publications were English-language observational studies reporting CKD testing or screening, laboratory identification of CKD, recorded diagnosis, monitoring, nephrology referral, treatment patterns or clinical and economic outcomes associated with CKD care. Economic evaluations of CKD screening in the general population were also eligible. Experimental studies and randomized controlled trials were not included. One reviewer screened all records identified through the structured Embase search and conducted targeted screening of records identified through the supplemental searches.

### Data extraction and synthesis

Data on study design, setting, population, CKD definition and stage, measures of care, outcomes and effect estimates were extracted into structured evidence tables. Where applicable, the tables also report the study-specific denominator population, care process or exposure definition, outcome definition and assessment period and whether estimates were reported, calculated, crude or adjusted. Findings were synthesized narratively by stage of the CKD care pathway and evidence type. No meta-analysis was conducted because the studies were heterogeneous in population, definitions, comparisons and outcomes. Similarly, data from the identified studies may not be directly comparable, and no statistical comparisons were conducted across studies or CKD categories.

Evidence was analysed according to a predefined care-pathway framework (Figure 1): (1) risk-based testing or population screening; (2) documented CKD diagnosis or self-reported CKD awareness; (3) disease monitoring; (4) nephrology care; (5) treatment; and (6) clinical and economic outcomes. Within each component, evidence was considered according to whether it was descriptive, associational or model-based. Study-specific terminology, definitions, populations and assessment periods are reported alongside results throughout to provide relevant context for interpretation.

**Figure 1.**
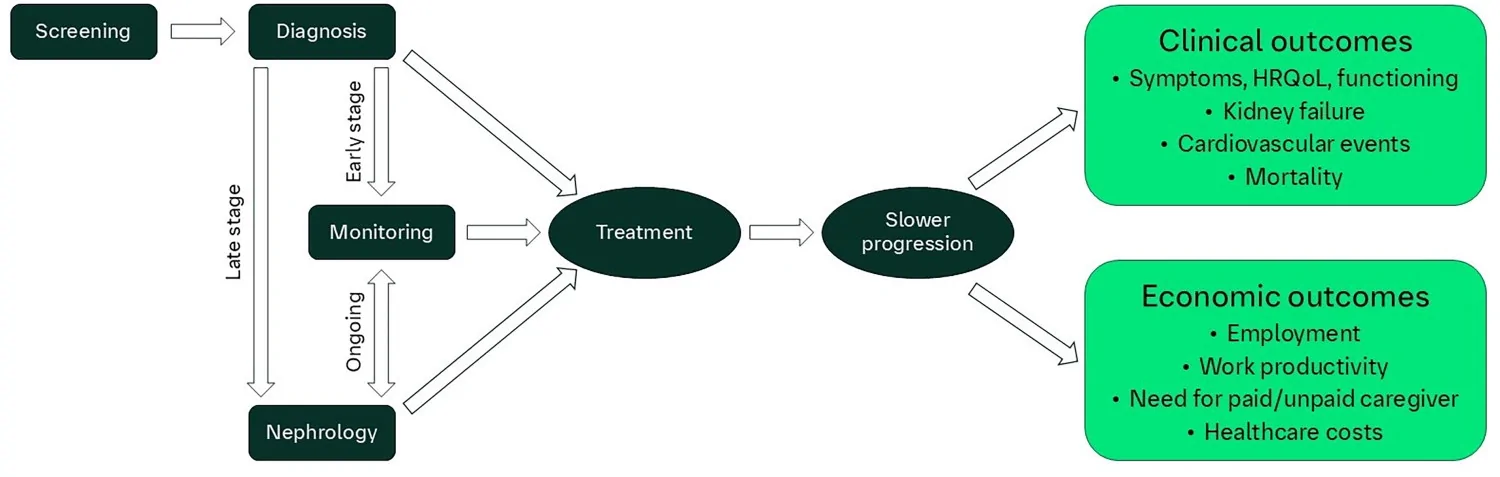
Proposed conceptual framework for gaps and potential intervention points across the CKD care pathway. *Note:* CKD: chronic kidney disease; HRQoL: health-related quality of life.

Given the targeted methodology of the review and the heterogeneity of the included evidence, no formal risk-of-bias or methodological-quality assessment was conducted. Study design and analytical characteristics, including whether estimates were adjusted, were nevertheless considered when interpreting the findings.

Overall, 35 studies were included, comprising 28 observational studies (in 29 publications) and seven economic evaluations. Study characteristics for economic evaluations and observational studies are provided in Table 6 and Supplementary Table 2, respectively. To attempt to mitigate limitations inherent to narrative reviews, findings have been compared with two identified systematic reviews on related topics.

**Table 1.** Kidney function and albuminuria testing among populations at increased risk of CKD.

Reference	Country (data source; study period)	At-risk population used as denominator	Testing definition and assessment period	Risk factor	N	Kidney function^a^ assessed, %	Albuminuria^a^ assessed, %	Both^a^ assessed, %	Either^a^ assessed, %
Jäger et al. [37]	Switzerland (FIRE database; 2013–2019)	Adults with diabetes, hypertension or established CVD with ≥18 months of follow-up after first identification of predisposing condition	Renal function assessment, defined as serum creatinine and/or albuminuria measurement within 18 months after first identification of the predisposing condition	DM	13,625	NR	NR	NR	76.2
				HTN	40,304	NR	NR	NR	66.7
				CVD	16,697	NR	NR	NR	69.5
Edmonston et al. [36]	US (PCORnet 2015–2020)	Adults aged 18–90 years with type 2 diabetes receiving active outpatient diabetes care without known CKD	Serum creatinine and UACR measurement within 15 months after the index visit; both measurements indicated full concordance and either measurement indicated at least partial concordance.	DM	316,234	80.3	26.0	24.9	81.4
Alfego et al. [34]	US (Labcorp; 2013–2018)	Adults with diabetes only and no CKD diagnosis code during the applicable study year	At least one eGFR result and at least one UACR or UPCR result during 2013–2018; complete CKD assessment required both components. Urine dipstick testing was excluded	DM only	4,567,453	83.6	32.2	28.7	NR
				HTN only	18,030,710	90.0	11.3	10.5	NR
				DM + HTN	5,679,730	93.3	43.0	41.4	NR
				DM and/or HTN	28,277,893	89.6	21.0	19.7	NR
Chu et al. [35]	US (Optum 2017–2018)	Adults with ≥2 outpatient visits with hypertension or diabetes with available eGFR; excluding ESKD or hospice care patients	At least one UACR test recorded during 2017–2018. Other measures of urinary protein, including UPCR, 24-hour urine testing and dipstick protein, were not assessed	DM only	6519	NA^b^	NA^b^	47.1^a^	NA^b^
				HTN only	141,601	NA^b^	NA^b^	5.1^a^	NA^b^
				DM + HTN	43,988	NA^b^	NA^b^	53.1^a^	NA^b^

*Note:* CKD: chronic kidney disease; CVD: cardiovascular disease; eGFR: estimated glomerular filtration rate; ESKD: end-stage kidney disease; NR: not reported; UACR: urine albumin-to-creatinine ratio; uPCR: urine protein-to-creatinine ratio; US: United States.

^a^ Kidney function assessment was based on serum creatinine and/or eGFR, whereas albuminuria assessment was based on the study-specific urine test indicated. ‘Both’ denotes assessment of kidney function and albuminuria. ‘Either’ denotes assessment of at least one of these components. Unless otherwise stated, values represent crude proportions of eligible patients with the specified test, reported in the source publication or calculated directly from reported numerators and denominators, with no adjustment or weighting applied.

^b^ Patients were required to have ≥1 assessment of eGFR to be eligible for this study.

**Table 2.** Proportion of people with lab-defined CKD who have a documented CKD diagnosis by sex.

Reference	Country (data source; study period)	CKD category	CKD population used as denominator	Definition and ascertainment period for documented CKD diagnosis	*N*	With documented CKD diagnosis, crude %			OR (95% CI) for lacking a documented diagnosis, women vs men	*p* Value
						Overall	Men	Women		
Lomstein et al. [85]	Greenland (national EMR; 2019–2021)	1–5	≥1 eGFR value <60 mL/min/1.73 m² and/or UACR >3 mg/mmol	≥1 recorded ICD-10 kidney disease diagnosis code	2157	3.5	4.9	2.3	2.22 (1.37–3.61)^a^	<.001
Jonsson et al. [41]	Iceland (EMR of all healthcare institutions; 2008–2016)	1–5, prevalent	At study entry: ≥2 eGFR values <60 mL/min/1.73 m² (≥90 days apart) and/or persistent proteinuria	≥1 recorded ICD-10 CKD diagnosis code at study entry	11,710	29.4	38.9	22.8	2.16 (1.99–2.34)^a^	<.001
		1–5, incident	During follow-up: ≥2 eGFR values <60 mL/min/1.73 m² (≥90 days apart) and/or persistent proteinuria	≥1 recorded ICD-10 CKD diagnosis code during follow-up	14,286	11.5	15.2	8.0	2.07 (1.86–2.30)^a^	<.001
Tangri et al. [40]	France (THIN, 2015–2020)	G3	≥2 consecutive eGFR values 30 to <60 mL/min/1.73 m² (90–730 days apart)	≥1 ICD-9 or ICD-10 CKD diagnosis code recorded at any time before or up to 6 months after the second qualifying eGFR measurement	92,930	4.5	5.4	3.7	1.31 (1.13–1.51)^b^	NR
	Germany (German Disease Analyser, 2015–2020)				253,083	15.7	18.2	13.9	1.29 (1.21–1.39) ^b^	NR
	Italy (IQVIA LPD, 2015–2020)				131,564	23.0	28.5	19.0	1.69 (1.63–1.76) ^b^	NR
	Japan (Japan RWD, 2015–2020)				65,675	7.9	9.3	6.4	1.46 (1.39–1.54) ^b^	NR
	US (LCED, 2015–2020)				302,164	38.4	45.9	32.3	1.59 (1.50–1.69) ^b^	NR
	US (TriNetX 2015–2020)				1,476,733	35.7	44.8	29.2	1.77 (1.73–1.80) ^b^	NR
Swartling et al. [39]	Sweden (SCREAM project; 2009–2017)	G3–G5	≥1 eGFR value <60 mL/min/1.73 m²	≥1 recorded ICD-10 CKD diagnosis code before or at cohort inclusion	57,880	5.0	6.9	3.4	1.82 (1.72–1.89)^c^	<.001

*Note:* CI: confidence interval; CKD: chronic kidney disease; eGFR: estimated glomerular filtration rate; EMR: electronic medical record; ICD: International Classification of Diseases; LCED: Linked Claims and Electronic Medical Records Data; LPD: Longitudinal Patient Database; NR: not reported; OR: odds ratio; RWD: real-world data; SCREAM: Stockholm Creatinine Measurements; UACR: urine albumin-to-creatinine ratio; US: United States.

^a^ Calculated from raw numbers of men and women with and without diagnoses to allow comparison with other studies.

^b^ ORs were reported adjusted estimates from logistic regression adjusted for baseline covariates.

^c^ Transformed from the reported adjusted OR for diagnosis in women vs men to allow comparison with other studies. Reported value was the odds of diagnosis, women vs men: 0.55 (0.53–0.58).

**Table 3.** Percentage of patients with CKD under the care of a nephrologist.

Reference	Country (data source; study period)	CKD population used as denominator	Definition and assessment period for nephrology care	Subgroup	Denominator, N	Stage 3a, %	Stage 3b, %	Stage 4, %	Stage 5 (without KRT), %
Prevalent CKD									
von Samson-Himmelstjerna et al. [43]	Germany (nationwide statutory claims; 2022)	Patients with a reconfirmed claims-based diagnosis of CKD in at least two quarters of 2022; stage assigned using ICD-10-GM codes	At least one ambulatory health claim from a physician designated as a nephrologist or practicing dialysis during 2022	NA	1,301,122	35.9^a^^,^^b^		64.8^b^	74.6^b^
Incident CKD									
Kampmann et al. [44]	Denmark (KidDiCo and Danish National Patient Registry; 2007–2013)	Patients with incident CKD G3–G5, defined by two eGFR measurements <60 mL/min/1.73 m² 3 to 12 months apart, with no normal eGFR measurement between; dialysis and kidney transplant recipients were excluded	Kidney-related primary or secondary ICD-10 diagnosis code indicating nephrology outpatient contact within 12 months after the CKD incidence date	NA	65,920	1.8^a^^,^^c^		10.8^c^	34.8^c^
				Men	2550	NR		15.5^c^	38.1^c^
				Women	2985	NR		7.1^c^	30.6^c^
Swartling 2022 [39]	Sweden (SCREAM project; 2009–2017)	Adult Stockholm residents with a first outpatient eGFR <60 mL/min/1.73 m² and no nephrologist visit before baseline; prior KRT was excluded	Completed first nephrologist visit during the 18 months after the index eGFR value	Men	217,341	2.8^b^	7.4^b^	16.4^b^	29.4^b^
				Women		1.2^b^	2.2^b^	9.5^b^	20.8^b^

*Note:* CKD: chronic kidney disease; eGFR: estimated glomerular filtration rate; ICD-10-GM: International Classification of Diseases, 10th Revision, German Modification; KidDiCo: Kidney Disease Cohort of Southern Denmark; KRT: kidney replacement therapy; NA: not applicable; NR: not reported; SCREAM: Stockholm Creatinine Measurements.

^a^ Data reported for patients with stage 3 CKD rather than stratified into stage 3a and 3b.

^b^ Crude percentages as reported in original study.

^c^ Crude percentages calculated by review authors using the reported numerators and denominators for stage 4, stage 5 and stages 3–5.

**Table 4.** Percentage of patients with CKD receiving guideline-recommended treatment.

Reference	Country (data source; study period)	Population	*N*	SGLT2i^a^	ACEI/ARB^a^
CKD overall					
Forbes et al. [86]	UK (Oxford-RCGP Primary Care Sentinel Cohort; 2022)	Adults aged ≥18 years with CKD meeting guideline indications for SGLT2i^b^	138,183	17.0%	NR
CKD with diabetes					
Lamprea-Montealegre et al. [87]	US (Veterans Health Administration; 2019–2020)	Patients with T2D and ≥2 primary care encounters during 2019–2020 with CKD, excluding eGFR <15 mL/min/1.73 m^2^ and KRT	424,680	12%	NR
Nicholas et al. [88]	US (CURE-CKD Registry, Providence and UCLA Health; 2019–2020)	Adults aged ≥18 years with diabetes and CKD, excluding eGFR <15 mL/min/1.73 m^2^ and KRT	39,158	6.0%	70.7%
Edmonston et al. [36]	US (PCORnet; 2019–2020)	Adults with T2D and CKD eligible for ACEI/ARB and SGLT2i therapy; albuminuria 200–5000 mg/g and most recent potassium ≤5 mEq/L	4215	2.9%	68.8%
Forbes et al. [86]	UK (Oxford-RCGP Primary Care Sentinel Cohort; 2022)	Adults aged ≥18 years with CKD meeting guideline indications for SGLT2i^b^	106,468	22.0%	NR
Shin et al. [89]	US (Optum Labs Data Warehouse, 28 health systems; 2022–2023)	Adults aged ≥18; excluded eGFR <20 mL/min/1.73 m² Subgroup: DM + CKD only, class 1a-indicated SGLT2i^c^	96,273	12.6%	56.5%
		Subgroup: DM, CKD, + HF, class 1a-indicated SGLT2i^c^	6343	12.7%	57.0%
		Subgroup: DM, CKD, + ASCVD, class 1a-indicated SGLT2i^c^	95,686	12.3%	62.8%
		Subgroup: DM, CKD, HF, + ASCVD, class 1a-indicated SGLT2i^c^	52,126	13.9%	64.3%
Lan et al. [90]	Australia (Western Australian Department of Health Hospital Morbidity Data Collection, ReDiCare project; 2020–2024)	Adults aged >18 years with T2D and CKD and hospital admissions, excluding eGFR <15 mL/min/1.73 m^2^ and KRT	2216	21.3%	56.1%
CKD without diabetes					
Forbes et al. [86]	UK (Oxford-RCGP Primary Care Sentinel Cohort; 2022)	Adults aged ≥18 years with CKD meeting guideline indications for SGLT2i^b^	31,715	0.01%	NR
Shin et al. [89]	US (Optum Labs Data Warehouse; 2022–2023)	Adults aged ≥18; excluded eGFR <20 mL/min/1.73 m² Subgroup: CKD only, class 1a-indicated SGLT2i^c^	30,998	1.2%	30.6%
		Subgroup: CKD + HF, class 1a-indicated SGLT2i^c^	3877	4.5%	53.3%

*Note:* ACEI: angiotensin-converting enzyme inhibitor; ADA: American Diabetes Association; AHA/ACC/HFSA: American Heart Association/American College of Cardiology/Heart Failure Society of America; ARB: angiotensin receptor blocker; ASCVD: atherosclerotic cardiovascular disease; CKD: chronic kidney disease; DM: diabetes; EASD: European Association for the Study of Diabetes; eGFR: estimated glomerular filtration rate; HF: heart failure; KDIGO: Kidney Disease: Improving Global Outcomes; KRT: kidney replacement therapy; NICE: National Institute for Health and Care Excellence; NR: not reported; SGLT2i: sodium-glucose cotransporter 2 inhibitor; UK: United Kingdom; UKKA: United Kingdom Kidney Association; US: United States.

^a^ All treatment estimates were reported directly by the included studies as crude, unadjusted proportions.

^b^ According to UKKA, KDIGO and/or NICE guidelines.

^c^ According to ADA, EASD, AHA/ACC/HFSA and/or KDIGO guidelines.

**Table 5.** Reported clinical outcomes according to CKD identification and care status.

Reference	Country (data source; study period)	Study population	Exposure or care-status comparison	Exposure definition and assessment period	CKD category	*N*	Clinical outcome	Results
Tangri et al. [42]	US (REVEAL-CKD, TriNetX; 2015–2020)	Adults aged ≥18 years with two consecutive eGFR values of 30 to <60 mL/min/1.73 m², 91–730 days apart. First recorded CKD diagnosis ≥6 months after second qualifying eGFR value	HR (95% CI) per 1 year of diagnostic delay	Diagnostic delay was the interval from 6 months after the second qualifying eGFR measurement to the first recorded ICD-9 or ICD-10 CKD diagnosis.	3	26,851	Kidney failure: kidney transplant or chronic dialysis identified using recorded procedure codes	1.63 (1.23–2.18)^a^
							MI and/or stroke identified using ICD-9 or ICD-10 codes	1.08 (1.03–1.13)^a^
							MI, stroke and/or HHF identified using ICD-9 or ICD-10 codes	1.08 (1.04–1.13)^a^
Molnar et al. [38]	Canada (Ontario Renal Reporting System, Ontario Health Insurance Plan; 2016–2019)	Adults initiating maintenance dialysis in Ontario from April 2016 through March 2019	Lack of timely CKD screening vs no identified screening care gap	*Lack of timely CKD screening:* Unknown nephrology referral eligibility 1–2 years before dialysis initiation together with ≥1 CKD risk factor (hypertension, diabetes or cardiovascular disease) >1 year before dialysis initiation *No identified care gap in CKD screening*: Earliest nephrologist visit <1 year before dialysis initiation, unknown nephrology referral eligibility 1–2 years before initiation and no CKD risk factors >1 year before initiation	Initiating maintenance dialysis	1850	All-cause mortality within 90 days after maintenance dialysis initiation.	16.8% vs 10.8%^b^
Lonnemann et al. [46]	Germany (German health insurance claims database; 2009–2012)	Adults aged >18 years with claims-coded CKD G1–G5 or dialysis during 2009–2012 and ≥1 documented G3–G5 or dialysis code. Timely- and late-referral cohorts were frequency-matched by age and sex.	Late vs timely outpatient nephrology referral	*Late referral:* Any of the following: (1) receiving dialysis but without nephrology care at study initiation; (2) no contact with a nephrologist throughout study (2009–2012); or (3) initial nephrology visit occurred in 2010–2012 and patient had stage 5 CKD or was receiving dialysis *Timely referral:* Any of the following: (1) CKD stage was available and patient was already receiving nephrology care at study initiation; or (2) initial nephrology visit occurred at any time during follow-up	3	11,861	Hospitalizations, PPPY	1.77 vs 0.95; *p* = .00003^c^
							Mortality	18.8% vs 6.7%; *p* = .0001^c^
					4	2746	Hospitalizations, PPPY	2.07 vs 1.35; *p* = .00001^c^
							Mortality	23.1% vs 12.6%; *p* = .006^c^
					5	706	Hospitalizations, PPPY	1.62 vs 1.16; *p* = .025^c^
					Dialysis	3371	Hospitalizations, PPPY	1.87 vs 1.69; *p* = .11^c^

*Note:* CI: confidence interval; CKD: chronic kidney disease; CVD: cardiovascular disease; eGFR: estimated glomerular filtration rate; HHF: hospitalization for heart failure; HR: hazard ratio; ICD: International Classification of Diseases; MI: myocardial infarction; PPPY: per patient per year; US: United States.

^a^ Results from Cox models adjusted for sex, age, history of type 1 or type 2 diabetes, number of pre-index visits, eGFR change rate and eGFR at the second qualifying eGFR measurement.

^b^ Percentages reported descriptively and were not adjusted for differences in patient characteristics or clinical trajectory. Statistical significance for this comparison was not reported in the original paper.

^c^ Rates and percentages were directly reported descriptive estimates from age- and sex-frequency-matched cohorts; no multivariable adjustment was applied to the reported rates or percentages.

**Table 6.** Cost-effectiveness of CKD screening in the general population.

Ref.	Country (perspective^a^; discount rate)	Screening^b^	Age	Screening interval	Treatment^c^	WTP threshold	Cost per QALY	WTP threshold status
Kairys et al. [61]	Germany (3.5%)	UACR (two tests at one follow-up)	Adults	2 years	None	NR	€3331.77	NR
							−€6175.89^b^	NR
Cusick et al. [62]	US (3%)	UACR (screening until age 75)	35	One time	None	$100,000; $150,000	$64,100	Below both
				One time	SGLT2i		$95,800	Below both
				10 years			$98,400	Below both
				5 years			$183,700	Above both
			45	One time	None		$55,600	Below both
				One time	SGLT2i		$92,800	Below both
				10 years			$93,100	Below both
				5 years			$153,300	Above both
			55	One time	None		$46,700	Below both
				One time	SGLT2i		$86,300	Below both
				10 years			$92,500	Below both
				5 years			$121,100	Above $100,000; below $150,000
			65	One time	None		$55,700	Below both
				One time	SGLT2i		$82,100	Below both
				10 years			$89,800	Below both
				5 years			$105,000	Above $100,000; below $150,000
			75	One time	SGLT2i		$82,200	Below both
Konta et al. [63]	Japan (2%)	UACR vs UPCR	60	Repeated (frequency NR)	None	¥5 million	¥1,966,433^c^	Below
					SGLT2i		¥1,765,599^c^	Below
Pouwels et al. [64]	Netherlands (costs 4%; health outcomes 1.5%)	At-home UACR	45–85	One time	SGLT2i, statin, diuretic, β-blocker, based on risk	€ 20,000	€9225.00	Below
			45–65				€7946.00	Below
			65–85				€10,310.00	Below
Zafarnejad et al. [65]	US (NR; 3%)	Cumulative sum statistic of eGFR	30	2 years	None	$50,000–$100,000	$21,680.06	Below
				1 year			$15,614.33	Below
			60	2 years			$49,792.88	Below
				1 year			$40,123.97	Below
			30 or 60	1 or 2 years	SGLT2i		Cost saving	
Wen et al. [67]	China (Societal; 3%)	UACR + SCr	45	One time	None	$35,501	$18,980	Below
				10 years			$15,541	Below
				5 years			$15,160	Below
				2 years			$12,452	Below
				1 year			$10,588	Below
			55	One time			$18,421	Below
				10 years			$18,607	Below
				5 years			$15,191	Below
				2 years			$13,881	Below
				1 year			$13,081	Below
			65	One time			$17,977	Below
				10 years			$16,861	Below
				5 years			$16,620	Below
				2 years			$13,431	Below
				1 year			$11,463	Below
Tangri et al. [66]	International, 31 countries (payer; specific to each country)	2 SCr tests	45	Annual for 10 years	None	Specific to each country		Below, except Saudi Arabia
			65					Below in all countries
		UACR + 2 SCr tests	45					Below in all countries
			65					Below in all countries

*Note:* ACEI: angiotensin-converting enzyme inhibitor; ARB: angiotensin receptor blocker; CKD: chronic kidney disease; KRT: kidney replacement therapy; QALY: quality-adjusted life-year; SCr: serum creatinine; SGLT2i: sodium-glucose cotransporter 2 inhibitor; UACR: urine albumin-to-creatinine ratio; UPCR: urine protein-to-creatinine ratio; US: United States; WTP: willingness-to-pay.

^a^ All studies used lifetime as the time horizon and the national healthcare system as the perspective unless otherwise stated.

^b^ Comparator is usual care unless otherwise stated.

^c^ All studies included ACEIs and/or ARBs in treatment received after screening. This column describes any additional treatments.

^d^ Includes cost of testing and treatment minus saved costs from averted/delayed KRT.

^e^ This study included only non-diabetic patients, rather than the general population. Because guidelines already indicate that screening is cost-effective and should be conducted in diabetic patients, this study of non-diabetic patients was included alongside the other studies of screening in the general population.

## Discussion

The findings are discussed according to the CKD care pathway, from patient identification and documented diagnosis through disease management and longer-term clinical and economic outcomes. Observational evidence identified gaps in risk-based testing, documented CKD diagnosis, disease monitoring, nephrology referral and treatment. These studies may provide insight into potential points of intervention to facilitate earlier treatment and improve long-term outcomes in patients with CKD, although the quantity and strength of the evidence varied across pathway stages and study designs and their relationships with treatment access and patient outcomes have not been causally established.

### Identifying patients with CKD in clinical practice

#### Receipt of guideline-recommended risk-based screening

Despite KDIGO recommendations for CKD testing in at-risk populations, current evidence suggests that some high-risk patients do not receive recommended kidney function and albuminuria testing. Across studies, albuminuria testing was consistently less frequent than serum creatinine or eGFR testing [34–37] (Table 1). Patients with multiple relevant comorbidities (e.g. both diabetes and hypertension) had higher reported rates of CKD testing, but albuminuria testing in particular was still rare [34,35].

One US-based study of 316,234 patients with type 2 diabetes (T2D) and without known CKD examined factors associated with nonconcordance with CKD testing recommendations, defined as receipt of neither serum creatinine nor albuminuria testing conducted in the 15 months following T2D index visit. Patients with some cardiovascular conditions, including coronary artery disease, peripheral arterial disease, hypertension and HF, were significantly less likely to be classified as nonconcordant with these recommendations (*p* < .001 for all). However, patients already receiving angiotensin-converting enzyme inhibitors (ACEIs), angiotensin receptor blockers (ARBs) or statins were significantly more likely to be classified as nonconcordant (*p* < .001 for all) [36].

In a population-based retrospective study of more than 9000 adults initiating maintenance dialysis in Ontario, Canada, study authors assessed potential gaps in care before dialysis initiation [38]. Among patients who had not seen a nephrologist or whose first nephrologist visit occurred less than a year prior to initiating dialysis, 62.1% had insufficient recorded serum creatinine or urine ACR measurements between 1 and 2 years before dialysis initiation to determine whether they had previously met the criteria for nephrology referral. Of these patients, 73.0% had at least one documented CKD risk factor, including hypertension, diabetes or cardiovascular disease and were classified by the study authors as having a ‘lack of timely CKD screening’. The findings therefore indicate insufficient recorded kidney testing within the predialysis care pathway among patients who subsequently initiated dialysis [38].

#### Reported proportions of documented CKD diagnosis and self-reported CKD awareness

The proportion of individuals with a documented CKD diagnosis varied substantially across study-defined CKD populations (Supplementary Table 3). Because the studies differed in population characteristics, healthcare settings, CKD definitions, outcome definitions and study periods, the estimates were not considered directly comparable and were not quantitatively pooled. However, to provide a visualization of the evidence, Figure 2 presents study-specific proportions of individuals with laboratory-indicated CKD who had a documented CKD diagnosis or, in one study, self-reported CKD awareness. The estimates tended to be higher in populations with more advanced CKD, but they varied widely within CKD categories and remained low in some advanced CKD populations. For example, one study reported that among the subgroup of patients meeting the KDIGO criteria for referral to a nephrologist, only 21.2% of men and 13.9% of women had a documented CKD diagnosis [39]. Among those who had previously been referred to a nephrologist, the corresponding proportions were higher but still suboptimal, at only 58.5% of men and 45.8% of women [39].

**Figure 2.**
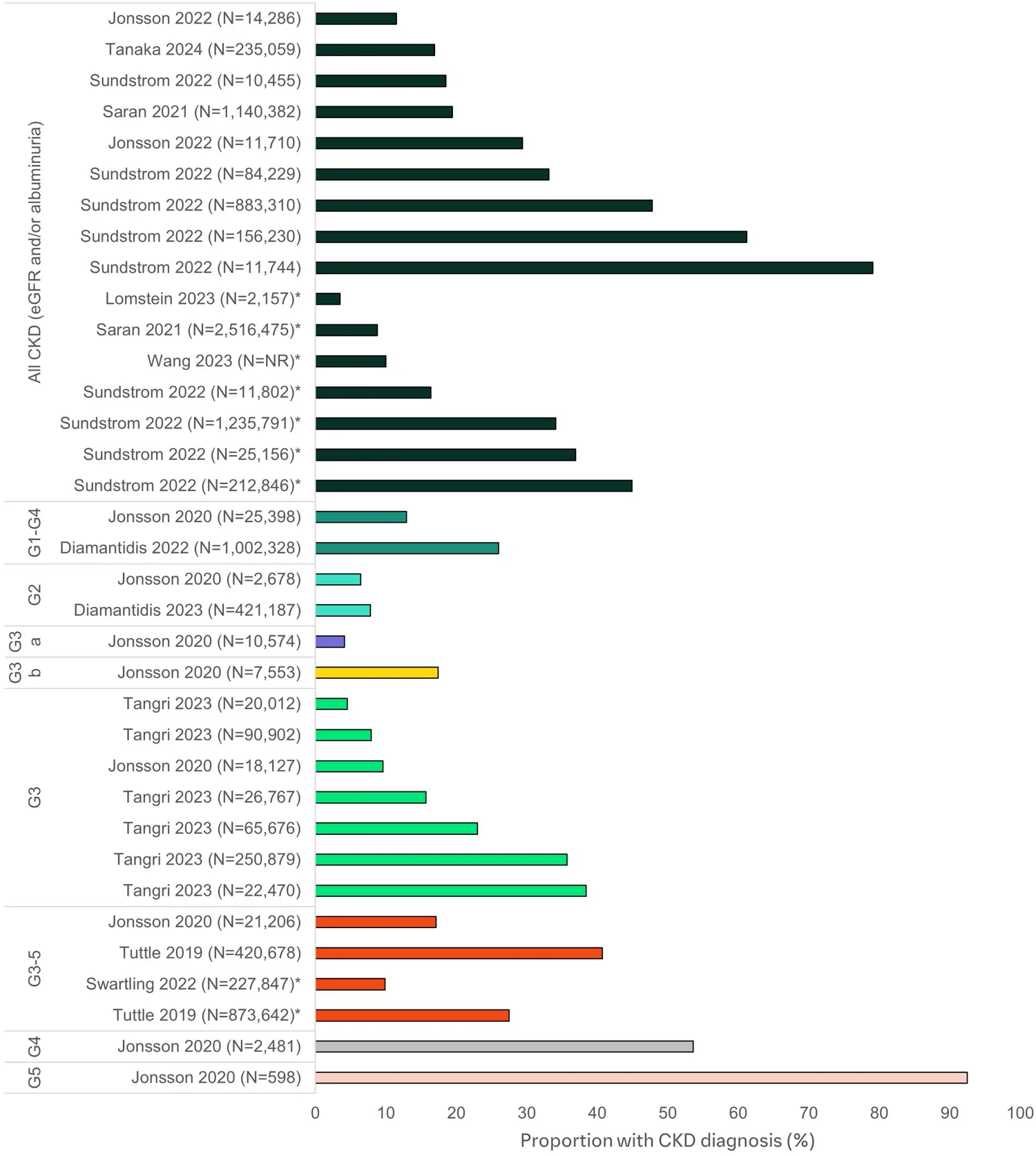
Reported CKD diagnosis rates by lab-determined CKD category. *Notes:* CKD: chronic kidney disease; eGFR: estimated glomerular filtration rate; *N*: total number of patients with documented diagnosis of CKD. The colours of the bars are intended to visually distinguish between the labelled CKD categories. Estimates were reported across heterogeneous populations, healthcare settings, CKD definitions, diagnostic definitions, study periods and denominators and should not be directly compared across studies or CKD categories. Except where noted, lab-determined CKD category required repeat testing (i.e., ≥2 qualifying lab values). The detailed study population, diagnostic definition and estimate corresponding to each plotted value are reported in Supplementary Table 3. *Repeated testing not required.

Although the drivers behind the heterogeneity in documented CKD diagnosis are not fully understood, some associated factors were identified. Three individual studies reported that patients with more advanced disease were more likely to have a documented CKD diagnosis [15,39,40]. Patients with relevant comorbidities such as hypertension [15,39,40], diabetes [15,39,40], HF [15,40] and CVD [39,40] were also more likely to have a documented CKD diagnosis, consistent with KDIGO guidelines [1]. In addition, studies consistently reported that women were significantly less likely than men to have a documented CKD diagnosis (Table 2). This association was consistent across subgroups defined by age [39,41], disease stage [39], albuminuria [39], presence of comorbidities [39] and receipt of care from a nephrologist [39]. Finally, two studies found that younger patients were significantly more likely than older patients to have a documented CKD diagnosis [39,40].

There is also limited evidence suggesting that substantial time may elapse between laboratory evidence of CKD and a documented CKD diagnosis. An analysis of a US database reported that the median time from laboratory values confirming stage G3 CKD (i.e. two eGFR measurements ≥30 and <60 mL/min/1.73 m^2^ taken 91–730 days apart) to a documented CKD diagnosis was nearly 5 years [40]. However, the median time to a documented diagnosis could not be determined for most study populations because fewer than 50% had a documented diagnosis during follow-up.

### Receipt of guideline-recommended disease management

Although identification and early management of CKD are often handled by primary care providers, later stages of disease should be managed by specialists. Both initiation of appropriate treatment and referral to nephrology require consistent monitoring of markers of kidney damage [1]. However, the available evidence suggests gaps in albuminuria monitoring, nephrology referral and receipt of guideline-recommended therapies.

#### Monitoring

Guideline-recommended CKD monitoring incorporates both eGFR, which assesses kidney function and urine ACR, which assesses kidney damage and independently contributes to CKD risk classification [1]. KDIGO Practice Points advise assessing GFR and albuminuria at least annually in people with CKD, with more frequent assessment in individuals at higher risk of progression when the results may affect therapeutic decisions. On repeat testing, changes exceeding expected biological and analytical variability, including *a* > 20% change in eGFR or a doubling of ACR, warrant further evaluation [1].

Inadequate or incomplete monitoring, particularly the omission of albuminuria assessment, may limit risk stratification and recognition of disease progression and may reduce opportunities to inform treatment and referral decisions. However, the direct impact of different monitoring frequencies or biomarker combinations on disease progression and clinical outcomes was not evaluated in the studies included in this review. Against these guideline recommendations, current evidence indicates that, although serum creatinine assessment was relatively common among patients with newly identified CKD, albuminuria assessment was substantially less common. In an analysis of quality indicators in Switzerland, 70.0% of stage G1–G4 patients received appropriate serum creatinine assessments within the first 18 months of laboratory evidence of CKD, but only 18.1% received an albuminuria assessment [37]. Similarly, a Swedish study of 227,847 adults found that only 34.0% of men and 27.5% of women received both re-measurement of creatinine and measurement of albuminuria in the 18 months after the first eGFR value <60 mL/min/1.73 m2 [39]. Men were significantly more likely to be monitored than women, and this monitoring occurred earlier; this was true across subgroups by age, disease stage, albuminuria and presence of comorbidities [39].

Delays in either documented CKD diagnosis or receipt of guideline-recommended nephrology care may be associated with differences in disease monitoring. The REVEAL-CKD study included patients with two consecutive eGFR values ≥30 and <60 mL/min/1.73 m2 taken 91 to 730 days apart [42]. This substudy focused on patients who did not have a documented CKD diagnosis for at least 6 months after the second eGFR measurement and compared disease monitoring in the 6 months before and after the documented diagnosis. Receiving a CKD diagnosis was associated with significantly increased rates of monitoring of systolic blood pressure (relative risk [RR]: 2.48; 95% confidence interval [CI]: 2.47–2.50), creatinine (RR: 2.35; 95% CI: 2.33–2.37), ACR (RR: 1.47; 95% CI: 1.30–1.66) and haemoglobin A1C (RR: 1.45; 95% CI: 1.42–1.49) [42]. Similarly, in a study of more than 1.3 million patients with stage 3 to 5 CKD, patients with at least one nephrology visit in the past year were significantly more likely to receive at least one assessment of creatinine, urinalysis and proteinuria quantification than those with no nephrology visit [43]. However, the studies identified in this review did not directly evaluate whether inadequate monitoring was associated with subsequent CKD progression, cardiovascular events, kidney failure or mortality, highlighting an important evidence gap.

#### Nephrology care

As expected, patients with more severe disease were most likely to have a recorded nephrologist visit (Table 3). While one study found that the majority of patients with stage 5 CKD had seen a nephrologist in the last year [43], only about one-third had a recorded visit with a nephrologist within the first 12 to 18 months following measurement of laboratory values that indicated incident stage 5 CKD [39,44]. Similarly, among patients initiating maintenance dialysis in the Canadian study who had sufficient historical laboratory data to assess prior eligibility for nephrology referral 31.8% were classified as having a late referral to nephrology^1^ [38]. Across identified studies, women and older patients were significantly less likely to have a recorded visit with a nephrologist [39,43,44].

#### Treatment

Across the included studies, many eligible patients with CKD did not receive guideline-recommended treatment with SGLT-2 inhibitors, and substantial proportions did not receive ACEIs/ARBs (Table 4). As anticipated, a documented CKD diagnosis was associated with improved rates of treatment. Patients in the REVEAL-CKD study had significantly higher prescription fill rates for ACEIs, ARBs, MRAs, SGLT-2 inhibitors and lipid-lowering medications in the 6 months after a documented CKD diagnosis compared to the 6 months before diagnosis [42]. In the subgroup of patients with comorbid T2D, prescription fill rates after documented CKD diagnosis were also higher for diabetes medications, including metformin, dipeptidyl peptidase-4 (DPP-4) inhibitors, sulphonylureas, SGLT-2 inhibitors, insulin and glucagon-like peptide-1 (GLP-1) receptor agonists [42].

### Long-term impact of inadequate disease management

#### Disease progression

The REVEAL-CKD study observed that a documented CKD diagnosis was significantly associated with a slowed decline of the mean annual eGFR, presumably because of improved access to appropriate monitoring and treatment [42]. In addition, a larger proportion of patients had stable eGFR (i.e. increase or no change) after diagnosis (46.6%) compared to before diagnosis (37.8%; *p* < .001), while a smaller proportion had rapid decline (i.e. annual eGFR decrease ≥4 mL/min/1.73 m^2^; 39.2% vs 47.1%; *p* < .001) [42]. In a retrospective study of more than 1 million Medicare Advantage patients with CKD identified using laboratory data, the authors compared annual eGFR decline between patients with vs without a documented CKD diagnosis code, described by the study authors as ‘clinical recognition’ [45]. In an adjusted model, those with clinical recognition had a significantly slower annual decline (*p* < .0001) [45].

Because of the progressive nature of CKD, the course of disease is affected not only by the presence of a clinical diagnosis, but by the time to documented diagnosis and therefore initiation of treatment. One year of diagnostic delay (i.e. time between second qualifying eGFR value and first recorded CKD diagnosis code) in patients with stage 3 CKD was associated with a significantly increased risk of progression to stage 4 or 5 CKD (hazard ratio [HR]: 1.40; 95% CI: 1.31–1.49) [42].

Similarly, available evidence suggests that timely receipt of care from a nephrologist was associated with significantly slower progression [46]. In a German study of patients with stage 3 to 5 CKD, the proportion of patients with stable disease (i.e. the disease stage was the same as the previous year) was calculated for each of the 3 years of follow-up. For all 3 years, patients with a timely referral^2^ to a nephrologist were significantly more likely to have stable disease than patients with a late referral^3^: 65.0% vs 52.0% (*p* = .009), 71.5% vs 60.3% (*p* = .049) and 72.9% vs 64.6% (*p* = .006) for follow-up years 1, 2 and 3, respectively.

In line with this evidence, Taiwan’s Ministry of Health and Welfare created the Early CKD Care Programme in an effort to slow the deterioration of kidney function and reduce the burden on the National Health Insurance [47]. The Early CKD Care Programme, which prioritizes active management of early stage (stages 1–3a) CKD, involves early referral to a nephrologist, medication management and a CKD case manager who provides nursing education and lifestyle consultations, routinely monitors kidney function and facilitates communication between doctors and patients. After adjusting for age, sex, baseline CKD stage and comorbidities, patients enrolled in the Early CKD Care Programme had significantly lower observed incidence of progression to stage 3b CKD over the median follow-up of 3 years (HR: 0.67; 95% CI: 0.55–0.81) [47].

#### Clinical outcomes

Evidence regarding the long-term outcomes associated with gaps in CKD testing, documented diagnosis and nephrology care was limited, and the included studies evaluated different populations, exposures and outcomes. Observational findings indicated that longer time to documented CKD diagnosis was associated with an increased risk of kidney failure, cardiac events and hospitalization [42], while timing of nephrology referral was associated with hospitalization and mortality [45] (Table 5). These observational associations do not establish that delayed diagnosis or nephrology referral caused the reported outcomes, but they may indicate a potential target for intervention.

Molnar et al. also reported unadjusted 90-day mortality of 16.8% among patients initiating maintenance dialysis who were classified as having a ‘lack of timely CKD screening’, compared with 10.8% among those with no identified screening care gap [38]. This descriptive comparison was not adjusted, statistical significance was not reported, and the analysis did not establish a causal relationship between the study-defined testing gap and the observed difference in mortality.

In addition, a study of general practitioners in England identified patients with CKD based on laboratory values (i.e. two measurements of eGFR <60 mL/min/1.73 m^2^ at least 90 days apart) and calculated the proportion who had Read codes identifying them as having CKD [48]. General practices were then divided into sextiles based on their CKD coding performance and, controlling for other patient- and practice-level factors, clinical outcomes were compared across sextiles. Compared with patients in average-performing practices, patients in the lowest-performing practices had significantly higher risks of cardiovascular hospitalizations (HR: 1.06; 95% CI: 1.02–1.11) and hospitalizations for HF (HR: 1.10; 95% CI: 1.01–1.19) than patients in the average-performing practices. Similarly, patients in the best-performing practices had significantly lower risk of cardiovascular hospitalizations (HR: 0.96; 95% CI: 0.92–0.99) and hospitalizations for HF (HR: 0.92; 95% CI: 0.86–0.99).

#### Healthcare costs

Data on the association between the impact of delayed or missing diagnosis and long-term healthcare costs were also sparse, with only one identified study directly comparing healthcare costs by diagnosis. Among patients with G4 to G5 CKD or kidney failure, patients with a previous, lower-stage diagnosis (i.e. ‘recognized’ CKD) had significantly lower total all-cause healthcare costs than those without a previous early-stage diagnosis (i.e. ‘unrecognized’) ($58,772 vs $67,137; *p* < .001) [49]. Patients with recognized CKD also had significantly lower CKD-related healthcare costs ($31,485 vs $33,794; *p* < .001).

Although only one study provided direct evidence regarding the association between delayed diagnosis and healthcare costs, other available evidence provides indirect support. One US-based study reported data on the costs of early-stage CKD (G2) during the year before and 2 years after study entry, stratified by the trajectory of kidney function [50]. Throughout the 3 years of the study, patients with the most rapidly declining kidney function consistently had the highest total medical costs, with mean annual costs ranging from $22,382 to $27,738, compared with $10,748 to $13,498 among patients with either slow eGFR decline or stable eGFR [50]. In another study, delayed nephrology referral^4^ was associated with significantly higher total medical costs than for timely referral^5^ for patients with stage 3 CKD (€13,054 vs €8149; *p* = .00008), patients with stage 4 CKD (€15,526 vs €10,953; *p* = .0002) and patients receiving haemodialysis (€26,747 vs €20,991; *p* = .002) [46]. Although a similar trend was seen in patients with stage 5 CKD, the difference was not statistically significant (€15,085 vs €12,634; *p* = .199) [46].

### Cost-effectiveness of CKD screening

A previously conducted systematic review reported that screening for CKD was cost-effective in selected populations with diabetes and/or hypertension [51]. These studies, as well as others, informed the KDIGO guideline recommending CKD screening in patients with these conditions [1]. A more recent systematic review (search conducted 2022) extended the assessment to other high-risk populations, including racial and ethnic groups at increased risk of CKD and explored the cost-effectiveness of CKD screening in the general population [52]. Based on the eight economic models identified [53–60], that systematic review concluded that CKD screening is not cost-effective in the general population. However, the long-term clinical and economic impact of screening projected in these models depended on assumptions regarding earlier diagnosis, monitoring, nephrology referral and treatment. As such, any changes in assumptions regarding treatment effectiveness, including the selection of clinical outcomes, can have a dramatic impact on model results. All eight economic models identified by the previous review were published in or before 2020 and therefore assumed that patients would be treated with ACEIs, ARBs and/or other antihypertensives, rather than newer, more effective treatments such as SGLT-2 inhibitors or nonsteroidal MRAs. Based on sensitivity analyses conducted in the original models, the authors noted that effectiveness of treatment was one of the most influential factors in determining the cost-effectiveness of screening [52]. In addition, few of those economic models included the incremental costs of cardiovascular outcomes among patients with CKD.

Since that systematic review was conducted, several economic models exploring the cost-effectiveness of CKD screening in the general population have been published (Table 6), with results varying across modelled strategies and willingness-to-pay (WTP) thresholds [61–67]. Although the models explored different countries, types of screening, screening frequency, patient populations and treatments utilized after diagnosis, several models projected that selected screening strategies were cost-effective at the applicable WTP threshold against which its findings were classified; however, some strategies exceeded the threshold and one study did not report a threshold. Despite the extensive heterogeneity, some trends were identified.

Consistent with the results of the previously conducted systematic review, the impact of treatment choice was reflected in newly identified models that directly compared different treatment options. One study reported that the addition of SGLT-2 inhibitors was estimated to be more cost-effective than ACEIs/ARBs alone [63], while another reported that adding SGLT-2 inhibitors to the model was associated with projected cost savings across age and frequency of screening [65]. In a third study, SGLT-2 inhibitors and ACEIs/ARBs alone were compared using cost-effectiveness acceptability curve proportions to identify preferred methods of screening and treatment by patient age categories (35, 45, 55, 65 and 75) and WTP thresholds of $100,000 per quality-adjusted life-year (QALY) and $150,000 per QALY [62]. Across all age groups and WTP thresholds, the preferred treatment was ACEIs/ARBs plus SGLT-2 inhibitors, rather than ACEIs/ARBs alone [62].

The heterogeneity of models in terms of region, healthcare settings, populations, screening methods and screening frequency makes it difficult to isolate the exact drivers behind the differences in results, and there may be multiple, interconnected drivers. However, both studies reporting that SGLT-2 inhibitors were estimated to be more cost-effective than ACEIs/ARBs alone incorporated the costs of cardiovascular events [63,65]; the study reporting that adding SGLT-2 inhibitors was cost-saving also included the costs of anaemia, a frequent complication of CKD [65]. These findings highlight the importance of appropriate treatment selection and ensuring that all relevant clinical outcomes are captured.

The effect of patient age on cost-effectiveness of screening varied somewhat across studies. Two of the identified studies reported that screening was estimated to be more cost-effective in younger patients [64,65]. In a study of one-time, at-home urine ACR (UACR) testing in the Netherlands, screening only younger individuals (aged 45–65) was estimated to be slightly more cost-effective than screening only older individuals (aged 65–85) (€7946/QALY vs €10,310/QALY) [64]. Similarly, a US-based model found that both annual and biennial screening using a cumulative sum statistic of eGFR calculated from a basic metabolic panel was estimated to be more cost-effective in patients aged 30 years compared to those aged 60 years [65]. However, a recent study in China found similar cost-effectiveness across age groups (patients aged 45, 55 or 65), although younger patients consistently had higher incremental QALYs than older patients [67]. In contrast, a US model found that it was usually more cost-effective to screen older patients [62]. Although the differences across studies make it difficult to determine the true underlying cause of these differences, the costs of cardiovascular events were incorporated into both models that showed screening is more cost-effective in younger patients [64,65], as well as the study in which cost-effectiveness was similar across age groups [67]; cardiovascular events were not included in the model that found screening was more cost-effective in older patients [62].

Only two studies directly compared different types of screening tests in the general population, limiting the ability to draw broad conclusions [63,66]. In a Japanese model of patients without diabetes, UACR and urine protein-to-creatinine ratio (UPCR) testing were both cost-effective at a WTP threshold of ¥5 million compared with no screening [63]. UACR was also found to be cost-effective compared with UPCR [63]. A multinational study compared two screening methods to current practice across 31 countries/regions: two eGFR assessments or two eGFR assessments plus one UACR measurement [66]. While both methods were consistently cost-effective compared with the country-specific WTP threshold, screening that included UACR assessment consistently had a lower incremental cost-effectiveness ratio [66].

Beyond the differences among the economic models, decisions regarding implementation of population-based screening require consideration of the characteristics of CKD, the potential benefits and harms of screening and the capacity of the healthcare system to provide confirmatory testing and subsequent care. CKD has several characteristics that could support screening, including a prolonged asymptomatic phase, the availability of established laboratory tests for detecting reduced kidney function and albuminuria and effective interventions that can reduce disease progression and related complications when initiated in appropriately selected patients.

However, the potential benefits of population-based CKD screening should be weighed against its limitations and possible harms. A single abnormal eGFR or albuminuria result may not indicate chronic disease; current KDIGO guidance recommends repeat testing after incidental detection of a low eGFR or elevated ACR and advises against assuming chronicity from a single abnormal result, because CKD generally requires evidence that the abnormality has persisted for at least 3 months [1]. One-time screening may therefore identify transient or nonpersistent abnormalities, potentially resulting in false-positive initial findings and additional confirmatory testing. Detection of abnormalities that would not otherwise become clinically consequential may also contribute to overdiagnosis, patient burden and increased use of primary care, laboratory and specialist services. Population-wide programs additionally incur testing, diagnostic follow-up, program-management and treatment costs and may have opportunity costs when staff, laboratory and financial resources are redirected from other healthcare activities [68,69]. Their value will therefore depend not only on test performance and modelled cost-effectiveness but also on the availability of clearly defined confirmatory pathways, capacity for follow-up, access to effective treatment, participation and uptake, equity and the healthcare setting in which screening is implemented [68–70].

Risk-based testing and population-based screening are supported by different types of evidence. Current KDIGO guidance advises testing people at risk of CKD using both GFR and UACR, and earlier economic evidence was generally favourable for selected populations with diabetes and/or hypertension [1,50,51]. However, the studies included in this review indicate that implementation remains incomplete, particularly for albuminuria assessment [34–37]. In contrast, the evidence identified for population-based screening consisted primarily of economic models, with findings varying according to the population, screening strategy and frequency, treatment pathway, model assumptions, healthcare setting and willingness-to-pay threshold [60–66]. These model-based projections do not establish the clinical effectiveness of implementing population-based screening programs. Although several models produced favourable estimates for selected strategies, the findings cannot support a single population-based screening approach across healthcare settings. Rather, the findings support efforts to improve delivery of guideline-recommended risk-based testing, while the value of extending screening to the general population remains dependent on the specific strategy and healthcare context.

## Conclusions

Despite the growing burden of CKD, current evidence indicates gaps in guideline-recommended risk-based testing, documented CKD diagnosis, disease monitoring, nephrology care and treatment. Limited observational evidence also suggests associations between some of these gaps and less favourable kidney and cardiovascular outcomes, although causality has not been established. Despite the elevated risk of CKD among patients with diabetes and/or hypertension, some patients in these high-risk groups did not receive recommended kidney function and albuminuria testing, with albuminuria assessment being particularly infrequent. Although the proportions with a documented CKD diagnosis were higher among patients with relevant comorbidities, they were still generally low, particularly for early-stage CKD.

Because the reported proportions of patients with documented CKD were generally low, using existing laboratory data may provide an additional estimate of the incidence and prevalence of CKD; however, this method may still result in an underestimate of the true burden. Because serum creatinine and albuminuria tests are not currently recommended for population-wide screening efforts, patients with available laboratory data may have been referred for testing because of existing risk factors or early, nonspecific symptoms such as fatigue. Some patients with undiagnosed CKD may therefore still be excluded from these analyses and may be captured only through proactive, population-wide screening.

Although data are still emerging, the available evidence suggests that delayed or absent documented CKD diagnoses are associated with subsequent delays in disease monitoring, nephrology referral and treatment initiation. These gaps in care have, in turn, been associated with faster progression, worse health outcomes and increased healthcare costs. Despite these observed associations, earlier economic models reported that CKD screening is not cost-effective in the general population. However, those earlier models did not include newer, more effective treatments.

Multiple systematic reviews have found that SGLT-2 inhibitors are effective in slowing the progression of CKD, reducing cardiovascular events and reducing mortality in both diabetic and non-diabetic patients, including those who are already taking ACEIs and/or ARBs [71–80]. In addition, the efficacy of SGLT-2 inhibitors is similar across stages of CKD [75,79]. Earlier economic models also did not typically include cardiovascular events, which are associated with substantial clinical burden and healthcare costs among patients with CKD [22,24,25,81–83]. Cardiovascular events and their subsequent healthcare costs also increase with microalbuminuria, macroalbuminuria and later stages of CKD [22,24,81]. These observations suggest that earlier diagnosis and treatment that slows disease progression may help limit the clinical and economic burden of cardiovascular morbidity in CKD.

In contrast, several more recent models, most of which incorporated cardiovascular events and some of which included SGLT-2 inhibitors as a treatment option, generated favourable cost-effectiveness estimates for selected population-based CKD screening strategies [61–67]. Some models also projected improvements in patient outcomes such as kidney failure and cardiovascular outcomes, particularly under scenarios in which screening was implemented as part of a comprehensive care plan that included diagnosis, monitoring and nephrology referral. However, these projections depended on the modelled strategy, assumptions and healthcare setting and do not establish the effects of implementing population-based screening in clinical practice.

A systematic review, which involves more rigorous methodology for searching and screening studies for eligibility, would provide a stronger level of evidence. Due to the extensive and rapidly evolving literature on CKD, the present review utilized a combination of targeted database searches to identify studies focused on the outcomes of interest. Although this review sought to be comprehensive, it was not systematic, so it is possible that some relevant studies were not identified by the employed searching methods. In addition, we identified two recent systematic reviews on related topics: the cost-effectiveness of CKD screening [52] and the long-term impact of not identifying CKD patients [84]. For both, the recent advances in understanding the complex downstream consequences of undiagnosed CKD have resulted in an abundance of new research in a limited amount of time. The new cost-effectiveness research we identified provides evidence that would likely alter the conclusions of the systematic review on the cost-effectiveness of population-wide CKD screening [52]. Similarly, Groehl et al. were unable to identify any studies examining the outcomes associated with delayed or missing diagnoses; rather, all identified studies reported on the outcomes associated with delayed nephrology referral [84].

Despite an increase in studies assessing documented CKD diagnosis, this review was limited by the somewhat sparse evidence regarding the long-term clinical and economic outcomes associated with delayed or missing diagnoses. Relatively few studies reported on the association between disease management and disease progression (four studies), clinical outcomes (four studies) and healthcare costs (three studies). In addition to the small number of studies, each focused on different aspects of disease management (testing, diagnosis or nephrology referral) and assessed different outcomes (decline in eGFR, progression to higher stage of disease, kidney failure, cardiovascular events, mortality, healthcare costs), preventing comparisons across populations.

A further limitation is that the included studies did not permit systematic attribution of observed gaps to patient-, provider- or healthcare-system-level determinants. Potential contributing factors may operate at multiple levels, but these were not inferred. Accordingly, this review focuses on documenting where gaps were observed and the outcomes associated with them rather than assigning their causes.

The limited observational studies evaluating longer-term consequences generally reported less favourable outcomes among patients with delayed or missing diagnosis or suboptimal referral or management, although the populations, exposures, outcomes and analytical methods differed substantially.

The evidence synthesized in this review comprised observational studies and economic models, each with distinct interpretive limitations. Observational associations and descriptive comparisons may be affected by residual confounding, selection bias and misclassification and cannot establish causality. Findings from economic models are projections dependent on model structure, input data, assumptions, extrapolations and setting-specific willingness-to-pay thresholds, rather than observed effects of implementing a screening program. Findings were therefore interpreted according to study design and were not assigned a uniform level of certainty.

In addition to the data gaps surrounding long-term clinical and economic outcomes, most identified studies also focused on a single aspect of the complex identification and management of CKD, so there is extremely limited direct evidence on how each of these aspects interact. This review identified evidence that diagnosis [42] and nephrology referral [43] are both associated with improved monitoring, while documented CKD diagnosis is also associated with increased prescription fills [42]. However, only the REVEAL-CKD study explored the relationships between documented CKD diagnosis, monitoring, treatment and long-term outcomes [42]. To better understand these relationships and identify points of intervention, additional longitudinal studies are needed that investigate the interplay between risk-based testing, documented CKD diagnosis, monitoring, nephrology referral, treatment initiation and long-term clinical and economic outcomes. Focused reviews of individual components of the pathway may also provide greater depth regarding specific gaps and potential interventions to address those gaps.

Although novel, effective treatments have been developed, the CKD burden continues to increase globally. For patients to reap the full benefits of these treatments, substantial improvements are needed in risk-based testing, particularly albuminuria assessment, documented CKD diagnosis, disease monitoring and referral to nephrologists. To facilitate these improvements, action is required from multiple stakeholders, including clinicians (both general practitioners and nephrologists), payers and policy makers, across a variety of markets. If clinical guideline developers decide to recommend population-based screening, uptake of screening is likely to depend on payer coverage, patient education initiatives and buy-in from clinicians. Although KDIGO guidelines establish recommendations for risk-based testing, monitoring, nephrology referral and treatment, compliance with these guidelines is suboptimal. Clinicians may need better resources and tools to help ensure that they are able to follow KDIGO recommendations for managing CKD.

## Supplementary Material

CKD_review_manuscript_suppl_new(clean_version).docx

## Data Availability

Data sharing is not applicable to this article as no new data were created or analysed in this study.

## Previous presentations

Preliminary results from this study were presented at ISPOR EU 2025 in Glasgow.

## Notes

1 These patients were eligible for nephrology referral based on available laboratory data, but either they did not see a nephrologist or their earliest nephrologist visit was less than 1 year prior to initiating dialysis.

2 Any of the following: (1) CKD stage was available and patient was already receiving nephrology care at study initiation; or (2) initial nephrology visit occurred at any time during follow-up (2009–2012) while patient had stage 1–3 CKD.

3 Any of the following: (1) receiving dialysis but without nephrology care at study initiation; (2) no contact with a nephrologist throughout study (2009–2012); or (3) initial nephrology visit occurred in 2010–2012 and patient had stage 5 CKD or was receiving dialysis.

4 Any of the following: (1) receiving dialysis but without nephrology care at study initiation; (2) no contact with a nephrologist throughout the study (2009–2012); or (3) initial nephrology visit occurred from 2010 to 2012 and patient had stage 5 CKD or was receiving dialysis.

5 Any of the following: (1) CKD stage was available and patient was already receiving nephrology care at study initiation; or (2) initial nephrology visit occurred at any time during follow-up (2009–2012) while the patient had stage 1–3 CKD.
