# Supplementary material for: Delays in screening, diagnosis and monitoring are associated with worse clinical and economic outcomes for patients with chronic kidney disease: a narrative review
Source: Ann Med. 2026 Nov 11;58(1):2753927. doi: 10.1080/07853890.2026.2753927 (PMC13757860; doi:10.1080/07853890.2026.2753927)
Supplement: Supplementary file 1 [file IANN_A_2753927_SM4732.docx]

Delays in screening, diagnosis, and monitoring are associated with worse clinical and economic outcomes for patients with chronic kidney disease: A narrative review

Nicole Fusco^a^*; James Weatherall^b^; and Jayne Hurrell^c^

^a^Cencora, Conshohocken, PA, USA; ^b^Boehringer Ingelheim International GmbH, Ingelheim am Rhein, Germany; ^c^Cencora, London, UK

[Nicole.Fusco@Cencora.com](mailto:Nicole.Fusco@Cencora.com)

Delays in screening, diagnosis, and monitoring are associated with worse clinical and economic outcomes for patients with chronic kidney disease: A narrative review

# Supplementary materials

Supplementary Table 1. Study identification methodology

| Search method | Screening method | Source | Facet | Search strings |
| --- | --- | --- | --- | --- |
| Formal, structured search | Complete screening, 1 reviewer | Embase (via Ovid) | CKD | exp chronic kidney failure/ or exp diabetic nephropathy/ or (chronic kidney disease or chronic kidney failure or CKD or CKF or diabetic nephropathy or diabetic kidney disease or DKD or kidney disease).ti,ab. |
|  |  |  | Epidemiology (diagnosis, screening, incidence, prevalence, mortality) | exp incidence/ or exp prevalence/ or exp mortality/ or ((delay* or late*) adj2 (diagnos* or screen*)) or (inciden* or prevalen* or mortalit* or fatal* or death* or screening or screened or diagnosis or diagnostic rate* or underdiagnos* or undiagnos*).ti,ab. |
|  |  |  | Treatment | exp polypharmacy/ or (polypharmacy or (treatment* adj2 pattern*) or ((practice or treatment*) adj2 guideline*)).ti,ab. |
|  |  |  | Economic | exp cost/ or exp fee/ or exp expenditures/ or exp presenteeism/ or exp absenteeism/ or exp "cost of illness"/ or exp health care cost/ or exp medical leave/ or exp hemodialysis/ or exp peritoneal dialysis/ or (cost or costs or costing or fee or fees or budget* or expenditure* or "resource use" or resource utilization or health care utilization or health care utilisation or absenteeism or presenteeism or work loss or employment or retirement or sick leave or sick day* or medical leave or dialysis or hemodialysis).ti,ab. |
| Informal search | Targeted screening, 1 reviewer | NHS EED (via Ovid) | CKD | (chronic kidney disease or chronic kidney failure or CKD or CKF or diabetic nephropathy or diabetic kidney disease or DKD or kidney disease).ti,ab. |
|  |  | EconLit (via Ovid) | CKD | (chronic kidney disease or chronic kidney failure or CKD or CKF or diabetic nephropathy or diabetic kidney disease or DKD or kidney disease).ti,ab. |
|  |  | Embase (via Ovid) | Various, not recorded | Various, not recorded |
|  |  | Medline (via Ovid) | Various, not recorded | Various, not recorded |
|  |  | Google Scholar | Various, not recorded | Various, not recorded |
| Grey literature | NA | Websites of national bodies and professional associations | NA | NA |
| Citation checking | Complete screening, 1 reviewer | SLRs identified by any method | NA | NA |

Key: CKD – chronic kidney disease; NA – not applicable; NHS EED – National Health Services Economic Evaluation Database; SLR – systematic literature review.

Supplementary Table 2. Characteristics of included observational studies

| Reference | Population | Country (years) | Study design | Data source | N | CKD stage  N (%) | Age  mean (SD) | Male  % | Outcomes reported | Outcome definition |
| --- | --- | --- | --- | --- | --- | --- | --- | --- | --- | --- |
| Alfego 2021 [1] | US adults aged >18 years who underwent testing at a Labcorp facility and were considered at risk for CKD based on laboratory orders containing ICD-9 or ICD-10-CM codes for type 1 or type 2 diabetes and/or hypertension. Patients were excluded for a given year and subsequent years if a CKD-associated ICD code appeared on any order | US (2013-2019) | Retrospective | Labcorp | 28,277,893 | NA | 60.6 (14.8) | 46.4 | Screening rate of high-risk individuals | Presence of both eGFR and UACR results within a given year; UPCRs were also included as an acceptable alternative to UACR. Testing rate was defined as the percentage of those with both tests performed out of all considered at risk within a given year |
| Chu 2023 [2] | Adults aged ≥18 years with hypertension or diabetes who had ≥2 outpatient visits, identified from a 5% random sample of the Optum deidentified EHR dataset. Patients with ESKD (defined as previous receipt of dialysis or kidney transplant) and those receiving hospice care were excluded. Patients were required to have ≥1 eGFR assessment to be included | US (2007-2018) | Retrospective | NHANES; Optum EHR | 192,108 | NA | 60.3 (15.1) | 45 | Screening rate of high-risk individuals | Proportion of the study population who underwent ≥1 UACR. The study did not examine alternative measures, including UPCR, 24-hour urine albumin or protein quantification, or urine dipstick protein |
| Cleary 2022 [3] | Adults with CKD stages 3-5, defined as ≥2 eGFR measurements <60 mL/min/1.73 m² separated by ≥90 days, with the most recent measurement recorded within the preceding 2 years; patients with coded initiation of renal replacement therapy were excluded | England (2015-2016) | Retrospective | 637 general practices | 117,932 | 3-5: 100% | NR | NR | Impact of disease management | Practice-level CKD coding performance, defined as “the percentage of patients with CKD in a practice with a CKD stages 3-5 Read code.” The study assessed the association between CKD coding and subsequent clinical outcomes |
| Diamantidis 2022 [4] | Medicare Advantage enrollees with CKD stages 1-4 identified from the OptumLabs Data Warehouse between 2013 and 2018. Patients were required to have ≥2 serum creatinine values at least 90 days apart indicating CKD; those with eGFR <15 mL/min/1.73 m², ESRD, dialysis, or kidney transplant at baseline were excluded | US (2013-2018) | Retrospective | Medicare Advantage database | 1,002,328 | 1: 4,602 (0.5) 2: 650,275 (64.9) 3: 317,605 (31.7) 4: 29,846 (3.0) | 73.4 (7.9) | 42 | Documented CKD diagnosis | Clinical recognition through an ICD-9 585.x or ICD-10 N18.x code from 12 months before to 12 months after baseline |
|  |  |  |  |  |  |  |  |  | Impact of disease management | The study assessed whether CKD progression differed according to clinical recognition status; CKD progression was defined as “the rate of CKD progression as captured by eGFR decline over the 5-year study period” |
| Diamantidis 2023 [5] | MA enrollees aged ≥18 years with confirmed stage G2 CKD (2 consecutive eGFR values of 60-89 mL/min/1.73 m² at least 90 days and ≤15 months apart, second value between 2014 and 2017); ≥12 months of continuous MA enrollment before study entry; ≥1 medical claim; ≥1 subsequent eGFR value after study entry; ≥30 days of follow-up  Exclusion: Evidence of ESKD, dialysis, or kidney transplant in the 12 months prior to cohort entry | US (2014-2017) | Retrospective | Medicare Advantage database | 421,187 | G2: 100% | 72.6 (7.2) | 42.8 | Documented CKD diagnosis | Proportion of MA enrollees with laboratory-identified stage G2 CKD (2 consecutive eGFR values of 60-89 mL/min/1.73 m², ≥90 days apart) identified among all MA enrollees with serum creatinine data |
|  |  |  |  |  |  |  |  |  | Impact of disease management | CKD progression trajectories and associated costs: 5 distinct trajectories of eGFR change over time were identified using group-based trajectory modeling. Mean annual total healthcare costs (payer + out-of-pocket costs for inpatient, outpatient, pharmacy, and other services, inflation-adjusted to 2019 US dollars) were estimated for each trajectory group for 1 year before and 2 years after study entry |
| Edmonston 2024 [6] | Screening cohort: Adults 18-90 years with T2D, ≥2 outpatient visits in prior 12 months, no known CKD, receiving antidiabetic treatment, and eGFR ≥60 mL/min/1.73 m² | US (2015-2020) | Retrospective | PCORnet | 316,234 | NA | NR | 48.5 | Screening rate of high-risk individuals | Measurement of both serum creatinine (eGFR) and UACR within 15 months of the index visit. Measurement of either creatinine or UACR alone was considered partial concordance; no measurement of either was considered nonconcordant |
|  | **Treatment cohort:** Adults with T2D and CKD eligible for ACEI/ARB and SGLT2i therapy, receiving antidiabetic treatment, eGFR 30-90 mL/min/1.73 m², UACR 200-5000 mg/g, and serum potassium <5 mEq/L |  |  |  |  |  |  |  | Treatment | Prescription of ACEI or ARB and/or SGLT2i within 12 months before through 6 months after the index visit in patients with T2D, CKD, and albuminuria (UACR 200-5000 mg/g) and eGFR 30-90 mL/min/1.73 m² |
| Forbes 2024 [7] | Adults aged ≥18 years with CKD registered with practices in the Primary Care Sentinel Cohort of the Oxford-Royal College of General Practitioners  Research and Surveillance Centre database on December 31, 2022, who met ≥1 UKKA, KDIGO, and/or NICE indication for SGLT2i treatment. CKD was identified by CKD diagnostic concepts, ≥2 eGFR values <60 mL/min/1.73 m² ≥90 days apart, and/or ≥2 proteinuria measurements ≥90 days apart. Guideline-specific exclusions included type 1 diabetes, kidney transplant, and, for UKKA, polycystic kidney disease | UK (2022) | Cross-sectional | Primary Care Sentinel Cohort | 138,183 | NR | NR | 55 | Treatment | SGLT2i treatment: Proportion of patients meeting ≥1 guideline-directed indication who had been prescribed an SGLT2i based on the most recent information available before December 31, 2022 |
| Jager 2022 [8] | Patients aged ≥18 years from 483 general practitioners in German-speaking Switzerland with ≥1 consultation recorded. Two cohorts were defined: (1) Renal function assessment cohort: all patients with ≥18 months of follow-up after first evidence of any predisposing condition (diabetes, hypertension, or established CVD); (2) CKD care cohort: all patients with laboratory-confirmed CKD (≥2 eGFR <60 mL/min/1.73 m² and/or ≥2 UAC ≥20 mg/L and/or ≥2 ACR ≥3 mg/mmol at least 3 months apart), excluding those ever staged G5 | Switzerland (2013-2019) | Retrospective | FIRE database | Screening cohort: 47,201 CKD monitoring cohort: 14,627 | NR | NR | Screening cohort: 51.3 CKD monitoring cohort: 42.4 | Screening rate of high-risk individuals | Renal function assessment in patients with predisposing conditions (diabetes, hypertension, or established CVD): achievement of ≥1 measurement of serum creatinine and/or albuminuria within 18 months of first evidence of the predisposing condition. Achievement rate was defined as the ratio of patients with the quality indicator met to the total denominator population |
|  |  |  |  |  |  |  |  |  | Albuminuria monitoring | At least 1 assessment of albuminuria within 18 months from first laboratory evidence of CKD stage G1-4 |
| Jonsson 2022 [9,10] | Icelandic inhabitants aged ≥18 years with ≥1 serum creatinine measurement during 2008–2016. Prevalent CKD comprised individuals who met the study CKD definition at study entry. Incident CKD analyses included individuals without CKD at study entry who subsequently developed CKD during follow-up | Iceland (2008-2016) | Retrospective | EMR of all healthcare institutions in Iceland | 14,286 | 1: 1,762 (12.3) 2: 1,811 (12.7) 3a: 9,676 (67.7) 3b: 952 (6.7) 4: 68 (0.5) 5: 17 (0.1) | 69 (range: 18-106) | 47.8 | Documented CKD diagnosis | Number of individuals with a documented diagnosis code (N18 or N19) for pre-existing CKD and for incident CKD |
| Kampmann 2022 [11] | Patients with incident CKD G3-G5, defined by 2 eGFR measurements <60 mL/min/1.73 m² recorded at least 3 months and no more than 1 year apart, with no normal eGFR measurement between them; dialysis and kidney transplant recipients were excluded | Denmark (2007-2013) | Retrospective | KidDiCo | 5,535 | 4: 4,359 (78.8) 5: 1,176 (21.2) | NR | 46.1 | Nephrology referral | Proportion of incident CKD stage 4-5 patients (eGFR <30 mL/min/1.73 m²) not on dialysis or kidney transplant who had a kidney-related primary or secondary ICD-10 diagnosis code recorded within 12 months of CKD incidence date, used as a proxy for nephrology outpatient follow-up |
| Lamprea-Montealegre 2022 [12] | VHA patients with T2D and ≥2 primary care encounters during 2019-2020 who had CKD, defined as ≥2 outpatient eGFR values <60 mL/min/1.73 m² and/or ACR >30 mg/g obtained >90 days apart. Excluded hospice care, invalid date of birth or missing sex, eGFR <15 mL/min/1.73 m², chronic dialysis, and kidney transplant | US (2019-2020) | Cross-sectional | VHA | 424,680 | NR | NR | NR | Treatment | SGLT2i treatment: Any active prescription for empagliflozin, ertugliflozin, canagliflozin, or dapagliflozin from January 1, 2019-December 31, 2020 |
| Lan 2025 [13] | Adults aged >18 years with T2D and CKD admitted to a Western Australian quaternary hospital during January 2020-September 2024. CKD was defined by ICD-10-AM codes or eGFR <60 mL/min/1.73 m² for >3 months. Excluded any eGFR <15 mL/min/1.73 m², acute kidney injury, dialysis, kidney transplant, palliative care, discharge against medical advice, and death during hospitalization. For multiple admissions, only the last eligible admission was included | Australia (2020-2024) | Retrospective | ReDiCare project | 2,216 | NR | 78.2 (10.9) | 58.7 | Treatment | SGLT2i treatment and ACEI/ARB treatment: Prescription of the respective drug class at hospital discharge |
| Lomstein 2023 [14] | Greenlandic residents aged ≥20 years with a serum creatinine measurement within the preceding 24 months. The analysis included individuals classified as having CKD based on eGFR <60 mL/min or a combination of eGFR >60 mL/min and UACR >3 mg/mmol | Greenland (2014-2021) | Cross-sectional | Greenland EMR | 2,157 | 1: 668 (31) 2: 1,058 (49.05) 3a: 267 (12.38) 3b: 111 (5.15) 4: 46 (2.13) 5: 7 (0.32) | 61.2 (12.2) | 46.5 | Documented CKD diagnosis | Diagnosed CKD: Presence of ≥1 ICD-10 diagnosis code for CKD (DN00-08, DN17-19, DQ61, DZ49.0, DZ94.0, or DZ99.2) |
| Lonnemann 2017 [15] | Adults aged >18 years in the German health insurance claims database from the German Health Risk Institute with CKD stages 1-5 or dialysis during 2009-2012 and ≥1 documented CKD stage 3-5 diagnosis or dialysis during follow-up. Timely- and late-referral cohorts were frequency-matched by age and sex | Germany (2009-2012) | Retrospective | German Health Risk Institute | 42,048 | No CKD: 18,507 (44.0) CKD stage not specified: 2,087 (5.0) 1: 473 (1.1) 2: 2,254 (5.4) 3: 11,411 (27.1) 4: 2,636 (6.3) 5: 948 (2.3) Dialysis: 3,732 (8.9) | NR | 58.3 | Impact of disease management | Impact of timely vs late nephrology referral on CKD progression and clinical outcomes. Outcomes included all-cause hospital admissions per patient per year; all-cause mortality during the annual transition periods 2009-2010, 2010-2011, and 2011-2012; and annual progression to a more advanced CKD stage, dialysis, or death |
| Molnar 2023 [16] | Adults initiating maintenance dialysis in Ontario between April 1, 2016, and March 31, 2019. Excluded maintenance dialysis >90 days before the index date, no further evidence of dialysis >30 days after initiation, indicating potential recovery from acute kidney injury, or kidney transplant before dialysis initiation | Canada (2016-2019) | Retrospective | Ontario Renal Reporting System, Ontario Health Insurance Plan | 9,216 | Initiating maintenance dialysis: 100% | 66 (15) | 61.5 | Screening rate | Timely CKD screening care was assessed from kidney function information available 1-2 years before dialysis initiation and the presence of CKD risk factors >1 year before initiation |
|  |  |  |  |  |  |  |  |  | Nephrology referral | Timing of the earliest nephrologist visit and whether nephrology referral eligibility could be determined before dialysis initiation |
|  |  |  |  |  |  |  |  |  | Impact of disease management | All-cause mortality within 90 days after maintenance dialysis initiation, compared across the study-defined CKD care-gap groups |
| Nicholas 2023 [17] | Adults aged ≥18 years with diabetes and CKD identified during 2015-2020 in the CURE-CKD Registry and followed for ≥90 days during 2019-2020. CKD was defined by 2 measurements ≥90 days apart of eGFR <60 mL/min/1.73 m², UACR ≥30 mg/g, or UPCR ≥0.15 g/g, or an administrative CKD code with laboratory confirmation. Excluded eGFR <15 mL/min/1.73 m², dialysis, kidney transpl, and follow-up <90 days | US (2019-2020) | Retrospective | CURE-CKD Registry | 39,158 | G1 (with CKD): 4,970 (12.7) G2: 5,641 (14.4) G3a: 17,372 (44.4) G3b: 8,144 (20.8) G4: 3,031 (7.7) | 70 (14) | 50.4 | Treatment | Baseline prescribing of an ACEI/ARB and an SGLT2i based on EHR prescription records during 2019-2020; SGLT2i prescribing was evaluated among patients with mean baseline eGFR ≥30 mL/min/1.73 m² |
| Niu 2021 [18] | Inclusion: Adults aged ≥18 years, non-pregnant, with CKD stages I-IIIa (eGFR ≥90 mL/min/1.73 m² with signs of kidney damage [stage 1]; eGFR 60-89.9 mL/min/1.73 m² [stage 2]; eGFR 45-59.9 mL/min/1.73 m² [stage 3a]); ≥2 medical return visits between January 2012 and August 2017. Exclusion: Aged <18 years; pregnancy. After 1:2 propensity score matching (on age, sex, eGFR, and CKD stage), 3,114 patients were included (1,038 cases; 2,076 controls) | Taiwan (2012-2017) | Retrospective | Taipei Medical University Research Database | 3,114 | 1: 166 (5.3) 2: 1,216 (39.1) 3a: 1,732 (55.6) | 59.1 (16.1) | 56.3 | Impact of disease management | Risk of progression from CKD stage I-IIIa to CKD stage IIIb (eGFR <45 mL/min/1.73 m²) during the study period, comparing participants enrolled in the Early CKD Care Programme (case group) vs those receiving routine care (control group). Reported as crude and adjusted hazard ratios from Cox proportional hazards models |
| Saran 2021 [19] | All patients who appeared in the CDW Vital Status Files and the CDW Patient Tables and were alive during FY 2005 with ≥1 VA visit in the study year or the prior 2 years; enriched with new patients appearing in subsequent years. CKD identification based on eGFR (CKD-EPI equation from serum creatinine) and/or albuminuria/proteinuria assessment. Patients on KRT were analyzed separately | US (2014) | Cross-sectional | VA-REINS | 6,932,278 | 3a: 603,866 (8.71) 3b: 283,309 (4.09) 4: 86,805 (1.25) 5: 32,360 (0.47) | NR | 3a: 95.7 3b: 96.1 4: 96.7 5: 97.3 | Documented CKD diagnosis | Proportion of all VA users per FY identified as having CKD without KRT, estimated using 2 operational definitions: (1) Liberal definition: presence of ≥1 ICD-9-CM CKD-related diagnosis, ≥1 eGFR <60 mL/min/1.73 m², or ≥1 proteinuria measurement categorized as moderate or severe, at any time between FY 2006 and FY 2014. (2) Strict definition (KDIGO-aligned): ICD-9-CM CKD-related diagnosis, persistent eGFR <60 mL/min/1.73 m² (≥2 values ≥90 days apart), or persistent proteinuria (excluding urine dipstick alone) |
| Shin 2024 [20] | Adults aged ≥18 years engaged with 1 of 28 US health systems, with 6 months of continuous enrollment before April 1, 2022, and ≥1 outpatient serum creatinine measurement during April 1, 2021-March 31, 2022. Excluded eGFR <20 mL/min/1.73 m², maintenance dialysis, and kidney transplant. CKD was defined as eGFR <60 mL/min/1.73 m² or UACR ≥30 mg/g; when measured UACR was unavailable, values converted from UPCR or urine dipstick results were used | US (2022-2023) | Retrospective | Optum Labs Data Warehouse | 607,656 | NR | NR | 48.4 | Treatment | SGLT2i treatment: Presence of an outpatient medication order for canagliflozin, dapagliflozin, empagliflozin, or ertugliflozin, between April 1, 2022, and March 31, 2023   ACEI/ARB treatment: Presence of an outpatient medication order for a single or combination agent containing an ACEI or ARB during the same period |
| Spencer 2023 [21] | Adults aged ≥40 years with late-stage CKD (G4-G5) or ESKD identified in Optum Labs Data Warehouse. Required continuous medical and pharmacy coverage during baseline and follow-up. Excluded kidney transplant, hospice care, or ≥90 days long-term care during baseline | US (2011-2017) | Retrospective | Optum Labs Data Warehouse | 377,714 | G4-G5: 318,801 (84.4) Kidney failure: 58,913 (15.6) | NR | 45.2 | Impact of disease management | Healthcare costs associated with unrecognized progression to late-stage CKD or ESKD. Comparison of total all-cause and CKD-related costs during the first year following late-stage CKD/ESKD diagnosis between patients with and without prior CKD recognition |
| Sundström 2022 [22] | Adults identified from digital healthcare systems across 11 countries with CKD defined by laboratory criteria and/or CKD diagnosis code. Possible CKD defined as CKD diagnosis or 1 pathological eGFR/UACR value. | International (2018-2021) | Retrospective | Varies by country | 2.4 million | 3-5: 100% | Canada: 68 (17) Germany: 77 (11) Netherlands: 75 (12) Norway: 70 (16) Spain: 76 (14) Sweden: 68 (19) UK: 75 (14) | Canada: 51 Germany: 50 Netherlands: 45 Norway: 59 Spain: 52 Sweden: 52 UK: 42 | Documented CKD diagnosis | Proportion of patients with:  1) Possible CKD (defined as having a CKD diagnosis or 1 pathological UACR or eGFR value, where the chronicity of CKD was not confirmed);  2) Measured CKD (2 pathological UACR or eGFR values ≥90 days apart); and  3) Diagnosed CKD (a registered CKD diagnosis, with or without available pathological eGFR and/or UACR values) |
| Swartling 2022 [23] | Adult (aged ≥18 years) residents of Stockholm with ≥1 outpatient serum or plasma creatinine measurement corresponding to eGFR <60 mL/min/1.73 m² during 2009-2018. The first low eGFR was the index date. Individuals with a history of KRT or missing age or sex were excluded | Sweden (2009-2018) | Retrospective | SCREAM project | 227,847 | G3a: 181,540 (80) G3b: 34,207 (15) G4: 9,773 (4) G5 (no dialysis): 2,327 (1) | NR | 45 | Documented CKD diagnosis | Having ever received a CKD diagnosis was identified from administrative records since 1997; among those without a prior diagnosis, incident CKD diagnosis was assessed during 18 months of follow-up |
|  |  |  |  |  |  |  |  |  | Monitoring | Remeasurement of creatinine and measurement of albuminuria during the next 18 months |
|  |  |  |  |  |  |  |  |  | Nephrology referral | Having ever had a nephrologist visit was identified from administrative records since 1997; among those without a previous visit, incident nephrologist visit was assessed during 18 months of follow-up |
| Tanaka 2024 [24] | Adults ≥18 years with 2 eGFR results <90 mL/min/1.73m² obtained 90-360 days apart, with ≥360 days of prior records in the RWD database. Excluded age <18 years, insufficient look-back or death before follow-up | Japan (2004-2021) | Cross-sectional (repeated) | RWD database | 788,059 | G2, A1: 283,793 (36.0) G2, A2: 63,274 (8.0) G2, A3: 34,348 (4.4) G3a, A1: 31,316 (4.0) G3a, A2: 8,927 (1.1) G3a, A3: 9,010 (1.1) G3b, A1: 6,452 (0.8) G3b, A2: 3,035 (0.4) G3b, A3: 5,333 (0.7) G4, A1: 1,025 (0.1) G4, A2: 928 (0.1) G4, A3: 3,411 (0.4) G5, A1: 80 (0.0) G5, A2: 143 (0.0) G5, A3: 1,921 (0.2) | 63.9 (15.5) | 51.1 | Documented CKD diagnosis | Presence of ≥1 ICD-10 code for CKD between 360 days before and 90 days after the index date among patients meeting CKD criteria based on eGFR and/or proteinuria (D300, D41 [012], E0 [89] 2, E1 [013] [2], E748, I12, I13 [012], K767, M103, N0 [2-8], N13 [123], N14, N15 [089], N16, N18 [1-589], N19, N25, O10 [23], O12, Q61 [1-8], Q62 [0-3], R944) |
| Tangri 2023 [25] | Adults aged ≥18 years with 2 consecutive eGFR measurements of 30 to <60 mL/min/1.73 m², recorded 91-730 days apart during 2015-2020, and ≥12 months of continuous database presence before the first qualifying measurement. Included patients had no recorded CKD diagnosis before or within 6 months after the second qualifying eGFR measurement and subsequently received a CKD diagnosis ≥6 months later. Excluded solid organ transplant, KRT, or evidence of CKD stages 4-5 or kidney failure before the second qualifying measurement | International (2015-2020) | Retrospective | REVEAL-CKD Study (TriNetX only) | 26,851 | Stage G3: 100% | 71.3 (10.5) | 42.6 | Monitoring | Systolic blood pressure, creatinine, UACR, HbA1c, measurements during follow-up |
|  |  |  |  |  |  |  |  |  | Treatment | Recorded post-diagnosis medication prescription fill rates of CKD-relevant medications |
|  |  |  |  |  |  |  |  |  | Impact of disease management | Association between each additional year of CKD diagnostic delay and post-diagnosis clinical outcomes, including kidney failure (kidney transplant or chronic dialysis during follow-up, identified using recorded procedure codes: CPT, HCPCS, ICD-9-PCS or ICD-10-PCS), MACE (myocardial infarction and/or stroke), and MACE+ (myocardial infarction, stroke, and/or hospitalization for heart failure) |
| Tangri 2023 [26] | Adults aged ≥18 years with ≥2 consecutive eGFR measurements of ≥30 to <60 mL/min/1.73 m², >90 and ≤730 days apart, on or after January 1, 2015, and ≥12 months of continuous presence in the database before the first qualifying measurement. Patients with a recorded CKD diagnosis were retained and classified as diagnosed; those without one were classified as undiagnosed | International (2015-2020) | Retrospective | REVEAL-CKD Study: THIN cegedim (France), Disease analyzer (Germany), LPD (Italy), Japan RWD (Japan), LCED (US), TriNetX (US) | THIN cegedim: 20,012 Disease analyzer: 26,767 LPD: 65,676 Japan RWD: 90,902 LCED (US): 22,470 TriNetX (US): 250,879 | Stage G3: 100% | NR | THIN cegedim: 45.4 Disease analyzer: 41.9 LPD: 42.2 Japan RWD: 52.9 LCED: 44.7 TriNetX: 41.9 | Documented CKD diagnosis | Diagnosed stage 3 CKD: presence of an ICD-9 OR ICD-10 diagnosis code for CKD of any stage at any time before or up to 6 months after the second qualifying eGFR measurement (index); undiagnosed stage 3 CKD: absence of such a code during this period |
| Tuttle 2019 [27] | Adults with CKD in the CURE-CKD registry identified from EHRs. CKD defined by ≥2 measurements ≥90 days apart of eGFR <60 mL/min/1.73m², UACR >30 mg/g, and/or UPCR >150 mg/g, or CKD administrative codes. Excluded ESKD treated with dialysis or transplant | US (2006-2017) | Retrospective | CURE-CKD Registry | 606,064 | G1-G2: 137,784 (22.7) G3a: 226,693 (37.4) G3b: 100,239 (16.5) G4: 39,125 (6.5) G5 (no dialysis): 20,328 (3.4) Unknown: 81,895 (13.5) | NR | 44.1 | Documented CKD diagnosis | Presence of CKD administrative coding among patients meeting CKD laboratory criteria: CKD defined by ≥2 measurements ≥90 days apart of eGFR <60 mL/min/1.73 m² or by 1 eGFR measurement |
| von Samson-Himmelstjerna 2024 [28] | Patients covered by German statutory health insurance with ≥2 diagnoses of CKD stage 3-5 within 2022 (M2Q criterion). Analyses included patients alive through 2022 with follow-up claims | Germany (2020-2023) | Retrospective | German Statutory Health Insurance claims database | 1,301,122 | 3: 958,149 (73.6) 4: 207,043 (15.9) 5 (no KRT): 37,020 (2.8) 5 (with KRT): 98,910 (7.6) | NR | 53.3 | Monitoring | Monitoring of GFR and proteinuria measured by the number of quarters in 2022 with serum creatinine measurements (eGFR monitoring) and/or urinalysis; proteinuria quantification included UACR and/or total proteinuria measurement |
|  |  |  |  |  |  |  |  |  | Nephrology referral | Absolute count and the proportion of CKD patients who visited a nephrologist in ≥1 quarter in 2022. Referral to a nephrologist was defined as a health claim by an ambulatory nephrologist |
| Wang 2023 [29] | Nationally representative sample of Chinese adults aged ≥18 years from the Sixth China Chronic Disease and Risk Factor Surveillance (2018-2019) with eGFR and UACR measurements. | China (2018-2019) | Cross-sectional | China Chronic Disease and Risk Factor Surveillance Study | 176,874 | Stage 1-2: 73.3% Stage 3: 25.0% Stage 4-5: 1.8% | NR | NR | Documented CKD diagnosis | Awareness of CKD defined as self-reported diagnosed kidney disease among participants meeting CKD criteria (impaired kidney function and/or albuminuria). |

Key: ACEI – angiotensin-converting enzyme inhibitor; ACR – albumin-to-creatinine ratio; ARB – angiotensin receptor blocker; CKD – chronic kidney disease; CKD-EPI – Chronic Kidney Disease Epidemiology Collaboration; CPT – Current Procedural Terminology; CVD – cardiovascular disease; eGFR – estimated glomerular filtration rate; EHR – electronic health record; EMR – electronic medical record; ESKD – end-stage kidney disease; FY – fiscal year; GFR – glomerular filtration rate; HbA1c – hemoglobin A1c; ICD – International Classification of Diseases; KDIGO – Kidney Disease: Improving Global Outcomes; KRT – kidney replacement therapy; MA – Medicare Advantage; MACE – major adverse cardiac event; NA – not applicable; NHANES – National Health and Nutrition Examination Survey; NICE – National Institute for Health and Care Excellence; NR – not reported; RWD – real-world data; SCREAM – Stockholm Creatinine Measurements; SD – standard deviation; SGLT-2 – sodium-glucose cotransporter 2; T2D – type 2 diabetes; UACR – urine albumin-to-creatinine ratio; UK – United Kingdom; UPCR – urine protein-to-creatinine ratio; US – United States; VA – Veterans Affairs.

**Supplementary Table 3. Proportion of each study-defined CKD population with a documented CKD diagnosis or self-reported CKD awareness**

| **Reference** | **Country; data source; study period** | **CKD population used as denominator** | **Numerator type** | **Numerator definition** | **Denominator, N** | **Meeting the numerator definition, n (%)^a^** |
| --- | --- | --- | --- | --- | --- | --- |
| **All CKD stages, based on eGFR and/or albuminuria** | | | | | | |
| Jonsson 2022 [10] | Iceland; EMR of all healthcare institutions in Iceland; 2008-2016 | Adults in Iceland with prevalent (ie, present at study entry) CKD defined and staged according to KDIGO clinical practice guidelines, with either (a) ≥2 qualifying eGFR values (<60 mL/min/1.73 m²) ≥90 days apart, excluding serum creatinine within 10 days of an AKI episode, or (b) persistent proteinuria, defined as ≥2 of the following ≥90 days apart: UAER ≥30 mg/24 hours, UPER ≥150 mg/24 hours, UACR ≥30 mg/g, or urine dipstick value of 1+ or greater | Documented diagnostic code | CKD diagnosis code (ICD-10 N18 or N19) at study entry | 11,710 | 3,439 (29.4) |
| Jonsson 2022 [10] | Iceland; EMR of all healthcare institutions in Iceland; 2008-2016 | Adults who developed incident (ie, developed during follow-up) CKD defined and staged according to KDIGO clinical practice guidelines, with either (a) ≥2 qualifying eGFR values (<60 mL/min/1.73 m²) ≥90 days apart, excluding serum creatinine within 10 days of an AKI episode, or (b) persistent proteinuria, defined as ≥2 of the following ≥90 days apart: UAER ≥30 mg/24 hours, UPER ≥150 mg/24 hours, UACR ≥30 mg/g, or urine dipstick value of 1+ or greater | Documented diagnostic code | CKD diagnosis code (ICD-10 N18 or N19) at study entry | 14,286 | 1,639 (11.5) |
| Lomstein 2023 [14] | Greenland; EMR; 2014-2021 | Greenlandic residents aged ≥20 years with creatinine testing in the past 2 years and laboratory-identified CKD based on the most recent eGFR (<60 mL/min/1.73 m²) and/or UACR (>3 mg/mmol) value; chronicity was not confirmed by repeat testing | Documented diagnostic code | CKD diagnosis code (ICD-10 DN00-DN08, DN17-DN19, DQ61, DZ49.0, DZ94.0, or DZ99.2) | 2,157 | 75 (3.5) |
| Tanaka 2024 [24] | Japan; RWD database; 2004-2021 | Japanese adults meeting the study-defined CKD criteria based on eGFR and/or proteinuria. Participants had ≥2 eGFR measurements <90 mL/min/1.73 m² recorded 90-360 days apart; those classified as G2 without proteinuria or G2A1 were excluded from the CKD analysis | Documented diagnostic code | CKD diagnosis code (ICD-10 D300, D41 [012], E0 [89] 2, E1 [013] [2], E748, I12, I13 [012], K767, M103, N0 [2-8], N13 [123], N14, N15 [089], N16, N18 [1-589], N19, N25, O10 [23], O12, Q61 [1-8], Q62 [0-3], or R944) 360 days before to 90 days after index date | 235,059 | 39,788 (16.9) |
| Sundström 2022 [22] | Israel; country-specific digital healthcare system; 2018-2021 | Adults with possible CKD, defined as a registered CKD diagnosis or ≥1 pathological eGFR (<60 mL/min/1.73 m²) or UACR (≥30 mg/g) value; chronicity was not required | Documented diagnostic code | Registered CKD diagnosis (including ICD, ICPC, SNOMED, codes not specified) prior to index date, with or without available pathological eGFR and/or UACR values | 84,229 | 27,868 (33.1) |
| Sundström 2022 [22] | Canada; country-specific digital healthcare system; 2018-2021 | Adults with possible CKD, defined as a registered CKD diagnosis or ≥1 pathological eGFR (<60 mL/min/1.73 m²) or UACR (≥30 mg/g) value; chronicity was not required | Documented diagnostic code | Registered CKD diagnosis (including ICD, ICPC, SNOMED, codes not specified) prior to index date, with or without available pathological eGFR and/or UACR values | 1,235,791 | 421,795 (34.1) |
| Sundström 2022 [22] | Belgium; country-specific digital healthcare system; 2018-2021 | Adults with possible CKD, defined as a registered CKD diagnosis or ≥1 pathological eGFR (<60 mL/min/1.73 m²) or UACR (≥30 mg/g) value; chronicity was not required | Documented diagnostic code | Registered CKD diagnosis (including ICD, ICPC, SNOMED, codes not specified) prior to index date, with or without available pathological eGFR and/or UACR values | 25,156 | 9,293 (36.9) |
| Sundström 2022 [22] | Portugal; country-specific digital healthcare system; 2018-2021 | Adults with possible CKD, defined as a registered CKD diagnosis or ≥1 pathological eGFR (<60 mL/min/1.73 m²) or UACR (≥30 mg/g) value; chronicity was not required | Documented diagnostic code | Registered CKD diagnosis (including ICD, ICPC, SNOMED, codes not specified) prior to index date, with or without available pathological eGFR and/or UACR values | 11,802 | 1,932 (16.4) |
| Sundström 2022 [22] | Sweden; country-specific digital healthcare system; 2018-2021 | Adults with possible CKD, defined as a registered CKD diagnosis or ≥1 pathological eGFR (<60 mL/min/1.73 m²) or UACR (≥30 mg/g) value; chronicity was not required | Documented diagnostic code | Registered CKD diagnosis (including ICD, ICPC, SNOMED, codes not specified) prior to index date, with or without available pathological eGFR and/or UACR values | 212,846 | 95,575 (44.9) |
| Sundström 2022 [22] | Canada; country-specific digital healthcare system; 2018-2021 | Adults with measured CKD, defined as ≥2 pathological eGFR (<60 mL/min/1.73 m²) or UACR (≥30 mg/g) value recorded ≥90 days apart | Documented diagnostic code | Registered CKD diagnosis (including ICD, ICPC, SNOMED, codes not specified) prior to index date, with or without available pathological eGFR and/or UACR values | 883,310 | 421,795 (47.8) |
| Sundström 2022 [22] | Belgium; country-specific digital healthcare system; 2018-2021 | Adults with measured CKD, defined as ≥2 pathological eGFR (<60 mL/min/1.73 m²) or UACR (≥30 mg/g) value recorded ≥90 days apart | Documented diagnostic code | Registered CKD diagnosis (including ICD, ICPC, SNOMED, codes not specified) prior to index date, with or without available pathological eGFR and/or UACR values | 11,744 | 9,293 (79.1) |
| Sundström 2022 [22] | Portugal; country-specific digital healthcare data source; 2018-2021 | Adults with measured CKD, defined as ≥2 pathological eGFR (<60 mL/min/1.73 m²) or UACR (≥30 mg/g) value recorded ≥90 days apart | Documented diagnostic code | Registered CKD diagnosis (including ICD, ICPC, SNOMED, codes not specified) prior to index date, with or without available pathological eGFR and/or UACR values | 10,455 | 1,932 (18.5) |
| Sundström 2022 [22] | Sweden; country-specific digital healthcare data source; 2018-2021 | Adults with measured CKD, defined as ≥2 pathological eGFR (<60 mL/min/1.73 m²) or UACR (≥30 mg/g) value recorded ≥90 days apart | Documented diagnostic code | Registered CKD diagnosis (including ICD, ICPC, SNOMED, codes not specified) prior to index date, with or without available pathological eGFR and/or UACR values | 156,230 | 95,575 (61.2) |
| Saran 2020 [19] | US (VA-REINS; 2014) | US Veterans Health Administration users meeting the liberal operational CKD definition, based on ≥1 ICD-9-CM CKD code, ≥1 eGFR value <60 mL/min/1.73 m², or ≥1 moderate/severe proteinuria measurement at any time during FY 2006-FY 2014 | Documented diagnostic code | CKD-related diagnosis code (ICD-9-CM) | 2,516,475 | 220,666 (8.8) |
| Saran 2020 [19] | US (VA-REINS; 2014) | US Veterans Health Administration users meeting the strict operational CKD definition, based on an ICD-9-CM CKD code, ≥2 eGFR values <60 mL/min/1.73 m² ≥90 days apart, or quantitative moderate/severe proteinuria | Documented diagnostic code | CKD-related diagnosis code (ICD-9-CM) | 1,140,382 | 220,666 (19.4) |
| Wang 2023 [29] | China; Chronic Disease and Risk Factor Surveillance Study; 2018-2019 | Adults in a nationally representative Chinese survey with eGFR <60 mL/min/1.73 m² and/or UACR >30 mg/g at a single survey assessment | Self-reported CKD awareness | Self-reported previously diagnosed kidney disease, ascertained via survey | NR | NR (10.0)^c^ |
| **G1-G4** |  |  |  |  |  |  |
| Diamantidis 2022 [4] | US; Medicare Advantage database; 2013-2018 | MA enrollees with laboratory-confirmed CKD stages 1-4, defined by ≥2 serum creatinine measurements ≥90 days apart; patients with ESKD, dialysis, kidney transplant, or baseline eGFR <15 mL/min/1.73 m² were excluded | Documented diagnostic code | CKD diagnosis code (ICD-9 585.x or ICD-10 N18.x) recorded from 12 months before to 12 months after baseline | 1,002,328 | 260,467 (26.0) |
| Jonsson 2020 [9] | Iceland; EMR of all healthcare institutions in Iceland; 2008-2016 | Adults in Iceland with CKD defined and staged according to KDIGO clinical practice guidelines, with ≥2 qualifying eGFR values calculated from serum creatinine measurements ≥90 days apart, excluding serum creatinine within 10 days of an AKI episode | Documented diagnostic code | CKD diagnosis code (ICD-9 585-586.9 or ICD-10 N18-N19.9) during study follow-up | 25,398 | 3,282 (12.9)^b^ |
| **G2** |  |  |  |  |  |  |
| Diamantidis 2023 [5] | US; Medicare Advantage database; 2014-2017 | MA enrollees aged ≥18 years with confirmed stage G2 CKD, defined by ≥2 consecutive eGFR measurements of 60-89 mL/min/1.73 m² recorded ≥90 days and ≤15 months apart | Documented diagnostic code | CKD diagnosis code (ICD-9 585.x or ICD-10 N18.x) recorded at study entry | 421,187 | 32,666 (7.8) |
| Jonsson 2020 [9] | Iceland; EMR of all healthcare institutions in Iceland; 2008-2016 | Adults in Iceland with CKD defined and staged according to KDIGO clinical practice guidelines, with ≥2 qualifying eGFR values calculated from serum creatinine measurements ≥90 days apart, excluding serum creatinine within 10 days of an AKI episode | Documented diagnostic code | CKD diagnosis code (ICD-9 585-586.9 or ICD-10 N18-N19.9) during study follow-up | 2,678 | 172 (6.4) |
| **G3a** |  |  |  |  |  |  |
| Jonsson 2020 [9] | Iceland; EMR of all healthcare institutions in Iceland; 2008-2016 | Adults in Iceland with CKD defined and staged according to KDIGO clinical practice guidelines, with ≥2 qualifying eGFR values calculated from serum creatinine measurements ≥90 days apart, excluding serum creatinine within 10 days of an AKI episode | Documented diagnostic code | CKD diagnosis code (ICD-9 585-586.9 or ICD-10 N18-N19.9) during study follow-up | 10,574 | 438 (4.1) |
| **G3b** |  |  |  |  |  |  |
| Jonsson 2020 [9] | Iceland; EMR of all healthcare institutions in Iceland; 2008-2016 | Adults in Iceland with CKD defined and staged according to KDIGO clinical practice guidelines, with ≥2 qualifying eGFR values calculated from serum creatinine measurements ≥90 days apart, excluding serum creatinine within 10 days of an AKI episode | Documented diagnostic code | CKD diagnosis code (ICD-9 585-586.9 or ICD-10 N18-N19.9) during study follow-up | 7,553 | 1,311 (17.4) |
| **G3** |  |  |  |  |  |  |
| Jonsson 2020 [9] | Iceland; EMR of all healthcare institutions in Iceland; 2008-2016 | Adults in Iceland with CKD defined and staged according to KDIGO clinical practice guidelines, with ≥2 qualifying eGFR values calculated from serum creatinine measurements ≥90 days apart, excluding serum creatinine within 10 days of an AKI episode | Documented diagnostic code | CKD diagnosis code (ICD-9 585-586.9 or ICD-10 N18-N19.9) during study follow-up | 18,127 | 1,749 (9.6)^b^ |
| Tangri 2023 [26] | France; REVEAL-CKD, THIN Cegedim; 2015-2020 | Adults aged ≥18 years with 2 consecutive eGFR values 30 to <60 mL/min/1.73 m², >90 and ≤730 days apart | Documented diagnostic code | CKD diagnosis code (ICD-9 585.1-585.6, 585.9, 403, 403.01, 403.1, 403.11, 403.9, 403.91, 404, 404.01, 404.02, 404.03, 404.1, 404.11, 404.12, 404.13, 404.9, 404.91, 404.92, 404.93, 250.4, 250.41, 250.42, 250.43, 588, 588.1, 588.81, 588.89, or 588.9; ICD-10 N18-N18.6, N18.9, I12.0, I12.9, I13.0, I13.10, I13.11, I13.2, E10.2, E11.2, E11.21, E11.22, E11.29, N25.0, N25.1, N25.81, N25.89, or N25.9) any time before or up to 6 months after the second qualifying eGFR measurement | 20,012 | 892 (4.5) |
| Tangri 2023 [26] | Germany; REVEAL-CKD, German Disease Analyzer; 2015-2020 | Adults aged ≥18 years with ≥2 consecutive eGFR values 30 to <60 mL/min/1.73 m², >90 and ≤730 days apart. | Documented diagnostic code | CKD diagnosis code (ICD-9 585.1-585.6, 585.9, 403, 403.01, 403.1, 403.11, 403.9, 403.91, 404, 404.01, 404.02, 404.03, 404.1, 404.11, 404.12, 404.13, 404.9, 404.91, 404.92, 404.93, 250.4, 250.41, 250.42, 250.43, 588, 588.1, 588.81, 588.89, or 588.9; ICD-10 N18-N18.6, N18.9, I12.0, I12.9, I13.0, I13.10, I13.11, I13.2, E10.2, E11.2, E11.21, E11.22, E11.29, N25.0, N25.1, N25.81, N25.89, or N25.9) any time before or up to 6 months after the second qualifying eGFR measurement | 26,767 | 4,210 (15.7) |
| Tangri 2023 [26] | Italy; REVEAL-CKD, IQVIA LPD; 2015-2020 | Adults aged ≥18 years with ≥2 consecutive eGFR values 30 to <60 mL/min/1.73 m², >90 and ≤730 days apart | Documented diagnostic code | CKD diagnosis code (ICD-9 585.1-585.6, 585.9, 403, 403.01, 403.1, 403.11, 403.9, 403.91, 404, 404.01, 404.02, 404.03, 404.1, 404.11, 404.12, 404.13, 404.9, 404.91, 404.92, 404.93, 250.4, 250.41, 250.42, 250.43, 588, 588.1, 588.81, 588.89, or 588.9; ICD-10 N18-N18.6, N18.9, I12.0, I12.9, I13.0, I13.10, I13.11, I13.2, E10.2, E11.2, E11.21, E11.22, E11.29, N25.0, N25.1, N25.81, N25.89, or N25.9) any time before or up to 6 months after the second qualifying eGFR measurement | 65,676 | 15,129 (23.0) |
| Tangri 2023 [26] | Japan; REVEAL-CKD, Japan RWD; 2015-2020 | Adults aged ≥18 years with ≥2 consecutive eGFR values 30 to <60 mL/min/1.73 m², >90 and ≤730 days apart | Documented diagnostic code | CKD diagnosis code (ICD-9 585.1-585.6, 585.9, 403, 403.01, 403.1, 403.11, 403.9, 403.91, 404, 404.01, 404.02, 404.03, 404.1, 404.11, 404.12, 404.13, 404.9, 404.91, 404.92, 404.93, 250.4, 250.41, 250.42, 250.43, 588, 588.1, 588.81, 588.89, or 588.9; ICD-10 N18-N18.6, N18.9, I12.0, I12.9, I13.0, I13.10, I13.11, I13.2, E10.2, E11.2, E11.21, E11.22, E11.29, N25.0, N25.1, N25.81, N25.89, or N25.9) any time before or up to 6 months after the second qualifying eGFR measurement | 90,902 | 7,209 (7.9) |
| Tangri 2023 [26] | US; REVEAL-CKD, LCED; 2015-2020 | Adults aged ≥18 years with ≥2 consecutive eGFR values 30 to <60 mL/min/1.73 m², >90 and ≤730 days apart | Documented diagnostic code | CKD diagnosis code (ICD-9 585.1-585.6, 585.9, 403, 403.01, 403.1, 403.11, 403.9, 403.91, 404, 404.01, 404.02, 404.03, 404.1, 404.11, 404.12, 404.13, 404.9, 404.91, 404.92, 404.93, 250.4, 250.41, 250.42, 250.43, 588, 588.1, 588.81, 588.89, or 588.9; ICD-10 N18–N18.6, N18.9, I12.0, I12.9, I13.0, I13.10, I13.11, I13.2, E10.2, E11.2, E11.21, E11.22, E11.29, N25.0, N25.1, N25.81, N25.89, or N25.9) any time before or up to 6 months after the second qualifying eGFR measurement | 22,470 | 8,625 (38.4) |
| Tangri 2023 [26] | US; REVEAL-CKD Study, TriNetX; 2015-2020 | Adults aged ≥18 years with ≥2 consecutive eGFR values 30 to <60 mL/min/1.73 m², >90 and ≤730 days apart | Documented diagnostic code | CKD diagnosis code (ICD-9 585.1-585.6, 585.9, 403, 403.01, 403.1, 403.11, 403.9, 403.91, 404, 404.01, 404.02, 404.03, 404.1, 404.11, 404.12, 404.13, 404.9, 404.91, 404.92, 404.93, 250.4, 250.41, 250.42, 250.43, 588, 588.1, 588.81, 588.89, or 588.9; ICD-10 N18-N18.6, N18.9, I12.0, I12.9, I13.0, I13.10, I13.11, I13.2, E10.2, E11.2, E11.21, E11.22, E11.29, N25.0, N25.1, N25.81, N25.89, or N25.9) any time before or up to 6 months after the second qualifying eGFR measurement | 250,879 | 89,625 (35.7) |
| **G3-G5** |  |  |  |  |  |  |
| Jonsson 2020 [9] | Iceland; EMR of all healthcare institutions in Iceland; 2008-2016 | Adults in Iceland with CKD defined and staged according to KDIGO clinical practice guidelines, with ≥2 qualifying eGFR values calculated from serum creatinine measurements ≥90 days apart, excluding serum creatinine within 10 days of an AKI episode | Documented diagnostic code | CKD diagnosis code (ICD-9 585-586.9 or ICD-10 N18-N19.9) during study follow-up | 21,206 | 3,632 (17.1)^b^ |
| Swartling 2022 [23] | Sweden; SCREAM project; 2009-2018 | Adult (≥18 years) residents of Stockholm with ≥1 outpatient serum or plasma creatinine measurement corresponding to eGFR <60 mL/min/1.73 m² during 2009-2018 | Documented diagnostic code | CKD diagnosis code (ICD-10 E11.2, I13.1, I12.0, I13.2, N03.2-N03.7, N05.2-N05.7, N18.x, N19.x, N25.0, Z49.0-Z49.2, Z94.0, or Z99.2) before cohort inclusion or within 18 months of the first qualifying eGFR value | 227,847 | 22,564 (9.9)^d^ |
| Tuttle 2019 [27] | US; CURE-CKD Registry; 2006-2017 | Adults meeting the laboratory-based CKD criterion of ≥2 eGFR measurements <60 mL/min/1.73 m² recorded ≥90 days apart | Documented diagnostic code | CKD diagnosis code (ICD-9 or ICD-10) during study follow-up | 420,678 | 171,011 (40.7) |
| Tuttle 2019 [27] | US; CURE-CKD Registry; 2006-2017 | Adults meeting the laboratory-based CKD criterion of ≥1 eGFR measurement <60 mL/min/1.73 m² | Documented diagnostic code | CKD diagnosis code (ICD-9 or ICD-10) during study follow-up | 873,642 | 240,630 (27.5) |
| **G4** |  |  |  |  |  |  |
| Jonsson 2020 [9] | Iceland; EMR of all healthcare institutions in Iceland; 2008-2016 | Adults in Iceland with CKD defined and staged according to KDIGO clinical practice guidelines, with ≥2 qualifying eGFR values calculated from serum creatinine measurements ≥90 days apart, excluding serum creatinine within 10 days of an AKI episode | Documented diagnostic code | CKD diagnosis code (ICD-9 585-586.9 or ICD-10 N18-N19.9) during study follow-up | 2,481 | 1,330 (53.6) |
| **G5** |  |  |  |  |  |  |
| Jonsson 2020 [9] | Iceland; EMR of all healthcare institutions in Iceland; 2008-2016 | Adults in Iceland with CKD defined and staged according to KDIGO clinical practice guidelines, with ≥2 qualifying eGFR values calculated from serum creatinine measurements ≥90 days apart, excluding serum creatinine within 10 days of an AKI episode | Documented diagnostic code | CKD diagnosis code (ICD-9 585-586.9 or ICD-10 N18-N19.9) during study follow-up | 598 | 553 (92.5) |

^a^ Unless otherwise specified, all values are crude percentages, either as reported in the original publication or calculated directly from reported numerator (n) and denominator (N).

^b^ Jonsson 2020 [9] reported numerators and denominators for individual, mutually exclusive stages of CKD: G1, G2, G3a, G3b, G4, and G5. These were summed to calculate the numerators and denominators for groups of stages to provide estimates corresponding to those reported in other publications (eg, data for stage 3 was calculated by summing the numerators and denominators from stage 3a and stage 3b).

^c^ Weighted percentage; the corresponding unweighted numerator and denominator were not reported. Authors of the original publication reported that sample weights incorporated multistage sampling weight, the nonresponse weight, and the poststratification weight based on the sixth national census (2010) population of China.

^d^ Crude percentage, calculated by review authors using the reported denominator and a numerator calculated from subgroups reported in original publication. Of the 227,847 people included, 11,328 (5%) had a recorded CKD diagnosis code at cohort inclusion. An additional 7,088 men and 4,148 women received a recorded CKD diagnosis code within the 18 months following their first qualifying eGFR value (total numerator: 22,564).

Key: AKI – acute kidney injury; CKD – chronic kidney disease; eGFR – estimated glomerular filtration rate; EMR – electronic medical record; ESKD – end-stage kidney disease; FY – fiscal year; ICD – International Classification of Diseases; KDIGO – Kidney Disease: Improving Global Outcomes; KRT – kidney replacement therapy; LCED – Explorys Linked Claims and Electronic Medical Records Data; LPD – Longitudinal Patient Database; MA – Medicare Advantage; NR – not reported; RWD – Real World Data; UACR – urine albumin-to-creatinine ratio; UAER – urinary albumin excretion rate; UPER – urinary protein excretion rate; SCREAM – Stockholm Creatinine Measurements; VA – Veterans Affairs.

# References in supplementary materials

[1]	Alfego D, Ennis J, Gillespie B, et al. Chronic kidney disease testing among at-risk adults in the US remains low: real-world evidence from a national laboratory database. Diabetes Care. 2021;44(9):2025-2032.

[2]	Chu CD, Xia F, Du Y, et al. Estimated Prevalence and Testing for Albuminuria in US Adults at Risk for Chronic Kidney Disease. JAMA Network Open. 2023;6(7):e2326230-e2326230.

[3]	Cleary F, Kim L, Prieto-Merino D, et al. Association between practice coding of chronic kidney disease (CKD) in primary care and subsequent hospitalisations and death: a cohort analysis using national audit data. BMJ open. 2022;12(10):e064513.

[4]	Diamantidis CJ, Zepel L, Wang V, et al. Disparities in Chronic Kidney Disease Progression by Medicare Advantage Enrollees. American Journal of Nephrology. 2022;52(12):949-957.

[5]	Diamantidis CJ, Storfer-Isser A, Fishman E, et al. Costs Associated With Progression of Mildly Reduced Kidney Function Among Medicare Advantage Enrollees. Kidney Medicine. 2023;5(6):100636.

[6]	Edmonston D, Lydon E, Mulder H, et al. Concordance with screening and treatment guidelines for chronic kidney disease in type 2 diabetes. JAMA Network Open. 2024;7(6):e2418808.

[7]	Forbes AK, Hinton W, Feher MD, et al. Implementation of chronic kidney disease guidelines for sodium-glucose co-transporter-2 inhibitor use in primary care in the UK: a cross-sectional study. eClinicalMedicine. 2024;68.

[8]	Jager L, Rosemann T, Burgstaller JM, et al. Quality and variation of care for chronic kidney disease in Swiss general practice: A retrospective database study. PLoS ONE. 2022;17(8 August):e0272662.

[9]	Jonsson AJ, Lund SH, Eriksen BO, et al. The prevalence of chronic kidney disease in Iceland according to KDIGO criteria and age-adapted estimated glomerular filtration rate thresholds. Kidney International. 2020;98(5):1286-1295.

[10]	Jonsson AJ, Lund SH, Eriksen BO, et al. Incidence of and risk factors of chronic kidney disease: Results of a nationwide study in Iceland. Clinical Kidney Journal. 2022;15(7):1290-1299.

[11]	Kampmann JD, Heaf JG, Mogensen CB, et al. Referral rate of chronic kidney disease patients to a nephrologist in the Region of Southern Denmark: results from KidDiCo. Clinical Kidney Journal. 2022;15(11):2116-2123.

[12]	Lamprea-Montealegre JA, Madden E, Tummalapalli SL, et al. Prescription Patterns of Cardiovascular- and Kidney-Protective Therapies Among Patients With Type 2 Diabetes and Chronic Kidney Disease. Diabetes Care. 2022;45(12):2900-2906.

[13]	Lan NSR, McFadden B, Johnson A, et al. Real-World Prescribing of Renal and Cardiovascular Protective Drugs in Patients With Type 2 Diabetes and Chronic Kidney Disease: Analysis of Data From a Western Australian Quaternary Hospital. Heart, Lung and Circulation. 2025.

[14]	Lomstein FB, Kjaergaard M, Skovgaard N, et al. Reporting chronic kidney disease in Greenland. International Journal of Circumpolar Health. 2023;82(1):2261223.

[15]	Lonnemann G, Duttlinger J, Hohmann D, et al. Timely referral to outpatient nephrology care slows progression and reduces treatment costs of chronic kidney diseases. Kidney international reports. 2017;2(2):142-151.

[16]	Molnar AO, Nash DM, Emblem J, et al. Patient Care Gaps Prior to Maintenance Dialysis Initiation: A Population-Based Retrospective Study. Canadian Journal of Kidney Health and Disease. 2023;10:1-10.

[17]	Nicholas SB, Daratha KB, Alicic RZ, et al. Prescription of guideline-directed medical therapies in patients with diabetes and chronic kidney disease from the CURE-CKD Registry, 2019-2020. Diabetes, Obesity and Metabolism. 2023;25(10):2970-2979.

[18]	Niu S-F, Wu C-K, Chuang N-C, et al. Early Chronic Kidney Disease Care Programme delays kidney function deterioration in patients with stage I–IIIa chronic kidney disease: An observational cohort study in Taiwan. Bmj Open. 2021;11(1):e041210.

[19]	Saran R, Pearson A, Tilea A, et al. Burden and Cost of Caring for US Veterans With CKD: Initial Findings From the VA Renal Information System (VA-REINS) [Article]. American Journal of Kidney Diseases. 2021;77(3):397-405.

[20]	Shin J-I, Xu Y, Chang AR, et al. Prescription Patterns for Sodium-Glucose Cotransporter 2 Inhibitors in U.S. Health Systems. JACC. 2024;84(8):683-693.

[21]	Spencer D, Dunning S, McPheeters J, et al. Health Care Costs Associated With Unrecognized Progression to Late-Stage Kidney Disease. American Journal of Managed Care. 2023;29(2):E64-E68.

[22]	Sundström J, Bodegard J, Bollmann A, et al. Prevalence, outcomes, and cost of chronic kidney disease in a contemporary population of 2.4 million patients from 11 countries: The CaReMe CKD study. The Lancet Regional Health – Europe. 2022.

[23]	Swartling O, Yang Y, Clase CM, et al. Sex Differences in the Recognition, Monitoring, and Management of CKD in Health Care: An Observational Cohort Study. J Am Soc Nephrol. 2022;33(10):1903-1914.

[24]	Tanaka T, Maruyama S, Chishima N, et al. Population characteristics and diagnosis rate of chronic kidney disease by eGFR and proteinuria in Japanese clinical practice: an observational database study. Scientific Reports. 2024;14(1):5172.

[25]	Tangri N, Peach EJ, Franzén S, et al. Patient management and clinical outcomes associated with a recorded diagnosis of stage 3 chronic kidney disease: the REVEAL-CKD study. Advances in Therapy. 2023;40(6):2869-2885.

[26]	Tangri N, Moriyama T, Schneider MP, et al. Prevalence of undiagnosed stage 3 chronic kidney disease in France, Germany, Italy, Japan and the USA: Results from the multinational observational REVEAL-CKD study. BMJ Open. 2023;13(5):e067386.

[27]	Tuttle KR, Alicic RZ, Duru OK, et al. Clinical characteristics of and risk factors for chronic kidney disease among adults and children: an analysis of the CURE-CKD registry. JAMA Network Open. 2019;2(12):e1918169.

[28]	von Samson-Himmelstjerna FA, Steiger E, Kolbrink B, et al. Referral, monitoring, and factors associated with non-referral of chronic kidney disease in Germany: a nationwide, retrospective cohort study. The Lancet Regional Health–Europe. 2024;47.

[29]	Wang L, Xu X, Zhang M, et al. Prevalence of Chronic Kidney Disease in China: Results from the Sixth China Chronic Disease and Risk Factor Surveillance. JAMA Internal Medicine. 2023;183(4):298-310.
